# Outbreaks of Avipoxvirus Clade E in Vaccinated Broiler Breeders with Exacerbated Beak Injuries and Sex Differences in Severity

**DOI:** 10.3390/v14040773

**Published:** 2022-04-08

**Authors:** Ruy D. Chacón, Claudete S. Astolfi-Ferreira, Patrícia C. Pereira, Mario S. Assayag, Antony B. Campos-Salazar, David De la Torre, Lilian R. M. de Sá, Sonia R. Yokomizo de Almeida, Rose Elí Grassi Rici, Antonio J. Piantino Ferreira

**Affiliations:** 1 Department of Pathology, School of Veterinary Medicine, University of São Paulo, Av. Prof. Orlando M. Paiva, 87, São Paulo 05508-270, Brazil; ruychaconv@usp.br (R.D.C.); csastolfi@gmail.com (C.S.A.-F.); sergioassayag@uol.com.br (M.S.A.J.); daviddelatorreduque@gmail.com (D.D.l.T.); liliansa@usp.br (L.R.M.d.S.); 2 Inter-Units Program in Biotechnology, University of São Paulo, Av. Prof. Lineu Prestes, 2415, São Paulo 05508-270, Brazil; 3 Pole Alimentos Ltd., Uberlândia 38412-264, Brazil; patriciacalixtovet@yahoo.com.br; 4 Department of Neurobiology, Duke University, Duke University Road, Durham, NC 27708, USA; ac699@duke.edu; 5 LABIGEN, Laboratory of Molecular Biology and Genetics, Quito EC170521, Ecuador; 6 Department of Anatomy, Biomedical Sciences Institute, University of São Paulo, Av. Prof. Lineu Prestes, 2415, São Paulo 05508-270, Brazil; so36ma@usp.br; 7 Department of Surgery, School of Veterinary Medicine, University of São Paulo, Av. Prof. Dr. Orlando M. Paiva, 87, São Paulo 05508-270, Brazil; roseeli@usp.br

**Keywords:** *Avipoxvirus*, clade E, beak tropism, increased mortality, reduced hatchability, electron microscopy, molecular characterization

## Abstract

Avipoxvirus affects chickens and wild birds, and it is characterized by lesions on the nonfeathered parts of the body (the cutaneous form), or necrotic lesions in the upper respiratory tract (the diphtheritic form). In poultry farming, avian pox is usually controlled by live attenuated vaccines. However, there have been many reports of outbreaks, even in flocks of vaccinated birds. In the present study, different outbreaks of the emerging clade E avipoxvirus were detected in commercial breeder flocks of chickens vaccinated against fowlpox virus in Southeast Brazil. Clinical manifestations of these outbreaks included a marked prevalence of moderate to severe progressive lesions in the beaks of affected birds, especially in roosters with increased mortality (up to 8.48%). Also, a reduced hatchability (up to 20.77% fewer hatching eggs) was observed in these flocks. Analysis of clinical samples through light and transmission electron microscopy revealed the presence of Bollinger bodies and poxvirus particles in epithelial cells and affecting chondrocytes. PCR, sequencing, and phylogenetic analysis of major core protein (*P4b*) and DNA polymerase (*pol*) genes identified this virus as clade E avipoxvirus. We also developed qPCR assays for open reading frames (ORFs) 49, 114, and 159 to detect and quantify this emergent virus. These results show the arrival and initial spread of this pathogen in the poultry industry, which was associated with harmful outbreaks and exacerbated clinical manifestations in vaccinated commercial breeder flocks. This study also highlights the relevance of permanent vigilance and the need to improve sanitary and vaccination programs.

## 1. Introduction

The *Avipoxvirus* (APV) genus is in the subfamily *Chordopoxvirinae*, of the family *Poxviridae* [1]. It is characterized by a large double-stranded DNA genome in an oval or brick-shaped envelope. According to the International Committee on Taxonomy of Viruses [2], this genus is comprised of 12 accepted species: *Canarypox virus* (CNPV), *Flamingopox virus* (FLMPV), *Fowlpox virus* (FWPV), *Juncopox virus* (JNPV), *Mynahpox virus* (MYPV), *Penguinpox virus* (PNGPV), *Pigeonpox virus* (PGPV), *Psittacinepox virus* (PSPV), *Quailpox virus* (QUPV), *Sparrowpox virus* (SRPV), *Starlingpox virus* (SLPV), and *Turkeypox virus* (TKPV). However, new potential species are identified every few years [3,4,5].

APVs have a wide range of avian hosts and present variable levels of pathogenicity. APV strains are known to cause pox disease in at least 232 bird species and 23 orders [6]. Most of the APV strains are highly related to a limited range of specific hosts that suffer the main pox disease-associated pathologies. However, the possible infective range in susceptible hosts may be broader [7].

Infections can be classified as the cutaneous form (dry pox) with nodular proliferative lesions in nonfeathered areas of the body, such as combs, wattles, eyelids, legs, feet, and other parts of the head, or the diphtheritic form (wet pox) with fibronecrotic proliferative lesions in the mucous membrane of the upper respiratory tract, such as the mouth, esophagus, larynx, and trachea [1]. Systemic infections can sometimes be observed. The transmission of APV is caused by direct contact between birds through pecking, scratching, or exposed abrasions on the skin or by indirect contact, or through ingestion or inhalation of contaminated food or fomites. Additionally, some flying or crawling insects can act as mechanical vectors [8].

The severity of clinical manifestations depends on the initial virulence and pathogenicity of the causative APV strain. Additionally, intrinsic factors such as the age or breed of the bird can influence the severity of signs. Coinfections, particularly with immunosuppressive viruses, can exacerbate the clinical status [9], for example, genomic chimerism caused by the integration of the reticuloendotheliosis provirus into the APV genome [9,10].

Prevention of pox disease in commercial birds is mainly achieved via prophylactic vaccines and strict sanitary measures to avoid biological vectors and contaminated sources. Despite these efforts, there have been several reports of outbreaks worldwide [11]. These events cause a significant economic impact on poultry farming, associated with poor zootechnical performance indices and a drop in egg production, combined with costs of morbidity, mortality, flock renewal, and sanitation [1].

APV in wild and domestic birds persists due to the virus’s intrinsic tolerance of a wide range of environmental and climactic conditions over long periods of time. The presence of a photolyase confers the ability of DNA repair in APV strains [12]. The diagnosis of pox disease is confirmed by light and electron microscopy, serology, and molecular methods. Serological tests include virus neutralization, passive hemagglutination, immunodiffusion, immunofluorescence, immunoblotting, and enzyme-linked immunosorbent assay (ELISA). Genetic variability between APVs has mainly been studied in the context of DNA polymerase (*pol*) and major core protein 4b (*P4b*) genes. A phylogenetic analysis showed three major clades (A, B, and C), including seven, three, and one subclade(s), respectively [13].

The representative species of the genus, FWPW, is found in the subclade A1. Species PGPV, PNGPV, and TKPV, as well as species FLMPV, are contained in subclades A2 and A3, respectively, while CNPV and SLPV are correspondingly contained in subclades B1 and B2. Clade C contains the PSPV [13,14,15]. Clade D was proposed by an isolated study in Japanese quail [16]. The latest member, clade E avipoxvirus, was first reported in turkeys in Hungary [17], then in breeder chickens in Mozambique [18], and recently in backyard chickens in South Brazil [19].

The present study reports different outbreaks and describes phenotypic and genotypic characteristics of the emerging clade E avipoxvirus in vaccinated commercial breeder flocks in Southeast Brazil.

## 2. Materials and Methods

### 2.1. Description of Outbreaks and the Clinical Samples

**Figure 1:**
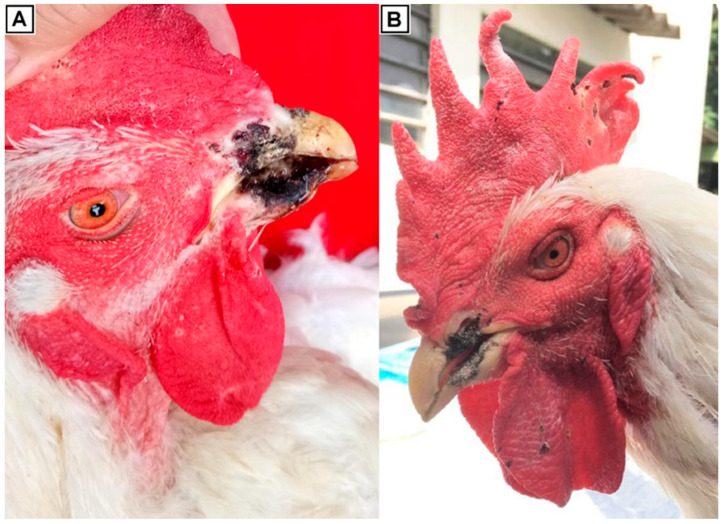
Gross pathology of affected roosters. (**A**) Major lesions consisted mainly of necrosis in nostrils and beaks. (**B**) Regular lesions in some birds included scabs and crusts in the combs and wattles.

These outbreaks occurred in three flocks of broiler breeders, one located in the Corumbataí municipality [22°13′12″ S, 47°37′33″ W], in the State of São Paulo, Brazil (Flock USP-1259), and two located in the Uberlândia municipality [18°55′08″ S, 48°16′37″ W], in the State of Minas Gerais, Brazil (Flocks USP-1408 and USP-1484) in the period between September 2018 and April 2019. Clinical samples from five birds per flock were sent to the Laboratory of Avian Diseases, University of São Paulo, Brazil. The main lesions were indicative of a poxvirus infection and consisted mainly of exacerbated scabs, crusts, and necrotic proliferative nodules emerging from nostrils and expanding to the beak (Figure 1A), and in extreme cases, loss of the upper part of the beak. Additionally, other birds presented less acute lesions, including small scabs in the combs, wattles, and eyelids (Figure 1B). The vaccination program included one or two steps of commercial fowlpox live vaccines when chicks were one day old and ten weeks old. A pool of injured pieces from the beak and skin from every flock was collected for microscopy. For molecular analysis, one sample for each outbreak was collected. Theses samples consisted of a pool from injured pieces from the beak collected from 5 different roosters from the same flock, as they exhibited the most prominent lesions.

### 2.2. Statistical Analysis of the Zootechnical Performance

A history of zootechnical performance was collected from two of the affected flocks (USP-1408 and USP-1484) during the period of egg production (weeks 25 to 65) (Tables S1 and S2). Descriptive analyses were performed and were compared with the estimated values according to the Ross 308 AP (AP95) lineage. We used the values from both flocks to calculate the observed weighted weekly egg laying performance and mortality indicators (Table S3). These discrete and/or continuous parameters included: (1) HDEP (hen-day production, denoted as a percentage of the daily egg quantity per total quantity of hens); (2) CEHP (cumulative eggs from hen production); (3) HE (hatching eggs, denoted as a percentage of the weekly hatched chicks per weekly incubated eggs); (4) CHM (cumulative hen mortality, culled or dead); and (5) CRM (cumulative rooster mortality, culled or dead).

The parameters were analyzed using SPSS 23 for MacOS (SPSS Inc., Chicago, IL, USA) and GraphPad Prism 7 (GraphPad Software Inc., La Jolla, CA, USA). The p-value threshold was set at 0.05. Longitudinal data from flocks included in the study were weighted in accordance with the number of birds in each flock. Time-matched estimated data were obtained from the performance standards guidelines for the lineage (Table S3). Data were pooled together in fixed time intervals (five weeks long) to compare the estimated and observed groups using two-way ANOVAs and Sidak’s multiple comparison test to compare across time intervals.

### 2.3. Histopathology and Electron Microscopy

Histopathology examination included pieces of beak and skin lesions. Samples were fixed in 10% neutral-buffered formalin and embedded in paraffin blocks. Sectional cuts were stained with conventional hematoxylin and eosin (H&E) to be analyzed by light microscopy.

Transmission electron microscopy (TEM) was performed on beak fragments. Samples were sectioned and fixed in modified Karnovsky solution (2.5% glutaraldehyde and 2% paraformaldehyde in 0.1 M sodium phosphate buffer solution with a pH of 7.4), according to Watanabe and Yamada [20] and Ciena et al. [21], postfixed in a 1% osmium tetroxide solution at 4 °C, and immersed in a 5% aqueous solution of uranyl acetate at room temperature. The sample was dehydrated in a series of solutions with increasing alcohol, immersed in propylene oxide, and incorporated into Spurr^®^ resin [22]. Semithin sections (350 nm) were collected on glass slides, stained with 1% toluidine blue, and analyzed using a Nikon Eclipse E-800 light microscope (Nikon Instruments Inc., Melville, NY, USA) to locate the desired region. Then, ultrafine cuts (60 nm) were performed and collected in 200 mesh copper grids (EMS^®^, Hatfield, PA, USA) in sequence, contrasted with 0.4% lead citrate and 4% uranyl acetate saturated solution [23]. The copper grids were examined using a Morgagni 268D transmission electron microscope at the Advanced Center for Diagnostic Imaging (CADI) at the Department of Surgery, School of Veterinary Medicine, University of São Paulo, São Paulo, SP, Brazil.

### 2.4. Nucleic Acid Extraction and PCR Detection of Avipoxvirus

DNA extraction of each sample was performed with a DNeasy^®^ Blood & Tissue kit (Qiagen, Hilden, Germany) according to the protocol for animal tissues recommended by the manufacturer. The DNA suspension was quantified with a NanoDrop One (Thermo Scientific^TM^, Wilmington, DE, USA) and stored at −80 °C for subsequent PCR procedures. Molecular detection of avipoxvirus was performed targeting the major core protein (*P4b*) and DNA polymerase (*pol*) genes with previously established primers and PCR procedures [13,24]. To exclude a possible coinfection with reticuloendotheliosis virus (REV), PCR targeting the long terminal repeats (LTRs) was performed [25]. A fowlpox commercial vaccine (Poximune^®^ AE, Ceva Animal Health, Lenexa, KS, USA) was used as a positive control for FWPV. Skin from specific-pathogen-free (SPF) chickens in 1.5 mL of phosphate-buffered saline (PBS) at pH 8.0 was used as a negative control.

### 2.5. Sequencing and Phylogenetic Analysis

PCR products from the major core protein (*P4b*) and DNA polymerase (*pol*) genes were purified with QIAquick^®^ Gel Extraction Kit (QIAGEN, Hilden, Germany). Sequencing was performed on a 3500xL Genetic Analyzer with the BigDye^TM^ Terminator v3.1 Cycle Sequencing Kit (Applied Biosystems, Carlsbad, USA). Sequence products were assembled with Geneious Prime^®^ 2020.2.4. (www.geneious.com) and aligned with MAFFT version 7 [26] along with other public avipoxvirus sequences retrieved from GenBank (https://www.ncbi.nlm.nih.gov/genbank/). The choice of the best fit substitution models and construction of phylogenetic trees were performed with MEGA version 7 [27]. Deduced amino acids were also aligned and analyzed to estimate the identity percentage.

### 2.6. Development of qPCR Assays for the Specific Diagnosis and Quantification of Clade E Avipoxvirus

An analysis of complete genome sequences corresponding to avipoxviruses was performed to identify exclusive sequence motifs for the clade E avipoxvirus. Seventeen available complete genome sequences of avipoxvirus strains were retrieved from GenBank [4,5,10,17,28,29,30,31,32] (Table S4). Genome alignment was performed with the iterative refinement method (FFT-NS-i) of MAFFT v7.45 [26]. Three genomic regions from the reference sequence for clade E avipoxvirus (GenBank: KP728110) were selected to design specific qPCR assays. Selected regions corresponded to open reading frames (ORFs) 49, 114, and 159. Two pairs of primers were designed per ORF (Table 1). The internal primers were designed to amplify an exclusive fragment for clade E avipoxvirus to be used in the specific qPCR assays. The external primers were based on the reference genome of clade E avipoxvirus (GenBank: KP728110) and were designed to amplify and clone a flanking fragment containing the template for internal primers. The formation of secondary structures (hairpin, self-dimer, and heterodimer) was analyzed with the NetPrimer tool (Premier Biosoft International, Palo Alto, CA, USA). Additionally, two pairs of primers (external and internal) were designed to amplify a fragment of the beta-actin gene (intron 4, GenBank: X00182) [33] and to quantify the number of *Gallus gallus* cells in each sample.

External primer products were amplified to a total volume of 25 μL, with 50 ng of template DNA corresponding to a sample from the last outbreak (USP-1484) to be used in the standard curve. PCR included 0.2 mM of each dNTP, 1.5 mM of MgCl2, 0.5 μM of each primer, 1× PCR buffer, and 0.625 U of Platinum Taq DNA Polymerase (Invitrogen, Carlsbad, CA, USA). The thermal conditions included an initial denaturation step at 94 °C for 3 min, followed by 36 cycles of 94 °C for 30 s, 60 °C for 30 s, and 72 °C for 1 min, and a final elongation step at 72 °C for 10 min. For the beta-actin amplification, all PCR conditions were maintained except for the annealing temperature, which was 55 °C. All external PCR products were visualized by 1.5% agarose gel electrophoresis (AGE) (Figure S1). External primer products were sequenced and used as a template for cloning to construct the standard curves for qPCR assays. The obtained sequences were submitted to GenBank under the accession numbers MW815506 (ORF49 template), MW815507 (ORF114 template), and MW815508 (ORF159 template).

For cloning, the PCR products from the external primers were purified with a QIAquick^®^ Gel Extraction Kit (QIAGEN, Hilden, Germany) and sequenced. Then, the fragments were cloned with the NEB^®^ PCR Cloning Kit into the pMiniT 2.0 vector with toxic minigene following a previous procedure [34] and plated with ampicillin at 37 °C overnight for selection (New England Biolabs, Ipswich, MA, USA). Plasmids were purified with the PureLink^TM^ Quick Plasmid Miniprep Kit (Thermo Fisher Scientific Baltics UAB, Vilnus, Lithuania), linearized with BamHI, and treated with RNAse A (Invitrogen, Carlsbad, CA, USA).

Standard curves were constructed with the cloned fragments of DNA. These were quantified with a Qubit^TM^ dsDNA BR Assay Kit in a Qubit 4 Fluorometer (Thermo Fisher Scientific Inc., Marsiling, Singapore). The calculation of the genome copies (GC) assumed that the average weight of a base pair (bp) was 650 Daltons and used Avogadro’s number. Thus, GC = [DNA concentration (ng) × 6.022 × 10^23^]/[DNA length (nt) × 10^9^ × 650]. Genome copies were adjusted to an initial concentration of 1 × 10^9^ GC/µL, and serial dilutions of 1:10 were prepared.

Quantitative PCR assays were conducted in a 20 μL reaction with 2 μL of template DNA, 0.6 µM of each primer, 10 μL of PowerUp™ SYBR^®^Green Master Mix (Applied Biosystems, Austin, TX, USA), and ultrapure DNase-free water. The thermal cycling program was configured using a fast method for the QuantStudio3 Real Time PCR System (Applied Biosystems, Marsiling, Singapore) according to the PowerUp™ SYBR^®^Green Master Mix (Applied Biosystems, Austin, TX, USA) manufacturer’s instructions. The initial temperature was 50 °C for 2 min, followed by 40 cycles at 95 °C for 1 s (for denaturation) and 60 °C for 30 s (for annealing and extension). Dissociation curves (melting) were plotted under conditions of 95 °C for 15 s, 60 °C for 1 min, and 95 °C for 15 s. All qPCR assays were conducted in triplicate for standard curve construction.

The analytical sensitivity was defined by the limit of detection (LoD) estimated by the lowest DNA template dilution that could be amplified by the qPCR assay, and the limit of quantification (LoQ) was estimated by the lowest DNA template dilution that could be quantified by the qPCR assay. The analytical specificity of the assays was tested by using a fowlpox commercial vaccine (Poximune^®^ AE, Ceva Animal Health, Lenexa, KS, USA) as a control.

Finally, qPCR assays (ORF49, ORF114, ORF159, B actin) were used to estimate the viral genome copy number per chicken cell (GC/cell). GC/cell was calculated as the ratio between the clade E avipoxvirus genome’s copy number per sample divided by the chicken genome’s copy number per sample (assumed as one beta-actin gene per chicken cell). Each value was estimated as the mean of three repetitions. With these ratios, the GC/cell range was indicated as the interval between the minimum value and the maximum value.

## 3. Results

### 3.1. Outbreaks and Clinical Samples

Pox disease outbreaks occurred in Southeast Brazil. Outbreak USP-1259 occurred in a breeder flock of approximately 48,000 birds with an initial sex ratio of 1 rooster to 10 hens (1:10). This flock received a single dose of a live fowlpox virus vaccine in the first week of life. Clinical signs presented at 36 weeks of age and continued until the moment of sampling (70 weeks of age). Outbreaks USP-1408 and USP-1484 belonged to the same company and occurred in different breeder flocks of 18,000 and 36,000 birds, respectively. Both flocks received two doses of a live fowlpox virus vaccine in the first and tenth weeks of life and had an initial sex ratio of 1:10 roosters to hens. Clinical manifestations started at 30 weeks of age and occurred until the end of the productive period (65 weeks of age).

Regarding the fowlpox virus vaccination, it was carried out using the wing web method. Evaluation of vaccination success was monitored by observing “takes” at the site where the vaccine was inoculated. “takes” consisted of visible lesions after scarification [1]. Vaccination records from these farms showed successful “takes” on 98% of the farm (including roosters and hens).

Regarding morbidity, affected birds account for approximately 45% of the total roosters and 20% of the total hens. Cumulative mortality data for roosters and hens are included in Tables S1 and S2. The most prominent manifestation in the three outbreaks was a lesion starting from the nostrils and extending to the beak, until the upper part of the beak was lost, particularly in roosters. In the case of hens, only the classical small scabs in the combs, wattles, and eyelids were observed. The birds had blackened, necrotic, crusted, proliferative, and progressive lesions in the upper and lower corneous beak at a range of severities. The lesions were bilateral and symmetrically distributed and exhibited erosion, ulceration, and necrosis with a partial loss of the culmen and gonys (Figure S2). Additionally, lesions extended to the hard palate and choanas (Figure S3). The dorsomedial caudal edges were covered by crusts, compromising the floor of the oral cavity. The ventromedial roof and edges of the upper beak were also compromised. At frontal cuts, necrosis and ulceration compromising part of the rostral nasal chamber was observed (Figure S3). The most severe cases involved the loss of portions (Figure 1A) or even most of the rostral part of the culmen and gonys. Occasionally, multiple foci of hemorrhagic crust were found in the combs and wattles (Figure 1B). Examination of the organs in the coelomic cavity revealed no changes in the lungs, air sacs, renal parenchyma, digestive, endocrine, reproductive system, or brain.

Different degrees of lesions on the corneous beak in roosters and breeding hens were detected (Figures S2 and S3). Figure S2A shows the initial manifestation, as a crack in the upper corneous beak, in the lateral posterior portion near the nostrils, exhibiting a serohemorrhagic crust. These lesions evolved to the culmen, extending along the rostral portions of the nostrils, and were covered by hemorrhagic crusts (Figure S2B), which increased as necrosis progressed (Figure S2C). The severity of lesions increased and extended from the nostrils, compromising the caudal edges of the culmen to the hard palate, choanas, and the floor and lateral caudal portion of the gonys (Figure S2D). Similarly, the ventromedial border showed erosion, loss of tissue, and hemorrhagic crust formation with bilateral symmetrical distribution (Figure S3A,B). The lesions in the nostrils progressed, causing internal damage to the choanas (Figures S3C–F).

### 3.2. Statistical Analysis of the Zootechnical Performance

**Figure 2:**
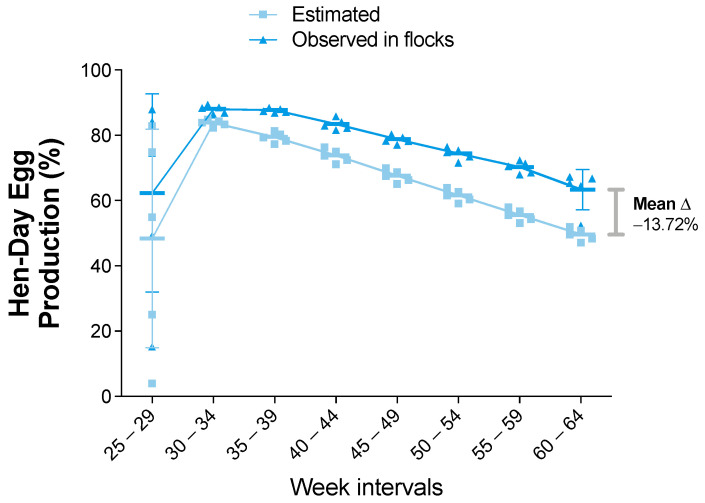
Percentages of hen-day egg production (HDEP) for estimated and observed flocks. Results are presented as mean and standard deviation, and mean differences (shown as delta “Δ” mean) were calculated subtracting observed from estimated values. Comparisons were performed using the two-way ANOVA test followed by the Bonferroni correction post-hoc test.

Descriptive analyses of the zootechnical indicators were performed and were compared with the “estimated” values according to the Ross 308 AP—P95 lineage (Table S3). First, the egg production indicators were analyzed. These indicators are one of the most relevant components in breeding farms. Good performance production was seen in the hen-day egg production curve (Figure 2). A gradual increase was observed until the 30–34-week interval, followed by a sustained decrease until the end of the productive period (week 64). As the hen-day egg production curve is a daily indicator, the comparisons throughout this period did not detect significant differences (*p* value > 0.05). However, the pooled data in the intervals showed a relatively higher production in the observed farms compared to the estimated data. In addition, a hen-day egg production curve mean difference of 13.72% at the end of the productive period was observed (Figure 2).

**Figure 3:**
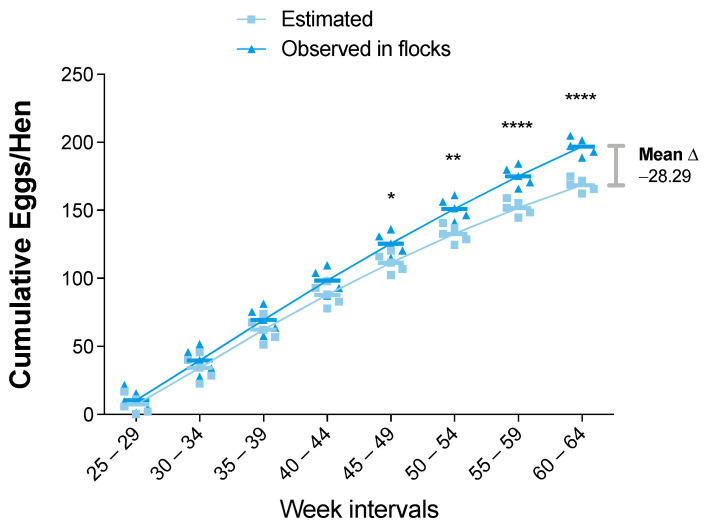
Cumulative egg/hen production (CEHP) for estimated and observed flocks. Results are presented as mean and standard deviation, and mean differences (shown as delta “Δ” mean) were calculated subtracting observed from estimated values. Comparisons were performed using the two-way ANOVA test followed by the Bonferroni correction post-hoc test. * *p* < 0.05; ** *p* < 0.005; *** *p* < 0.0005; **** *p* < 0.0001.

The cumulative eggs-hen production (CEHP) index showed a sustained increase in the observed data compared to the estimated data (Figure 3). However, the absolute difference between observed and estimated data increased over time and became statistically significant (*p* value < 0.05) from the 45–49-week interval onward. The CEHP mean difference was favorable to observed data, with 28.29 more eggs per hen than estimated at the end of the productive period (Figure 3).

**Figure 4:**
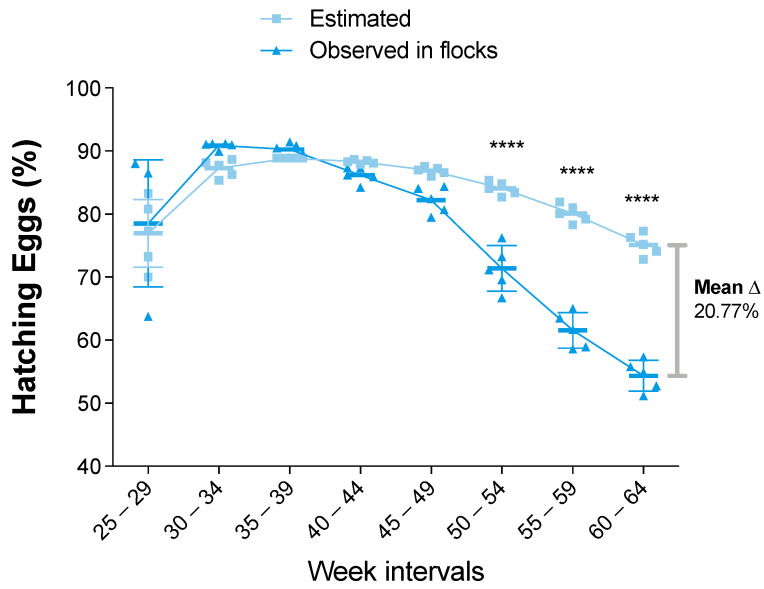
Percentages of Hatching Eggs (HE) for estimated and observed flocks. Results are presented as mean and standard deviation, and mean differences (shown as delta “Δ” mean) were calculated subtracting observed from estimated values. Comparisons were performed using the two-way ANOVA test followed by the Bonferroni correction post-hoc test. * *p* < 0.05; ** *p* < 0.005; *** *p* < 0.0005; **** *p* < 0.0001.

Up until this point, the data from the observed farms were equal to or better than the estimated data. However, a different pattern was observed in the hatching eggs (HE) comparison. As in the case of the previous indicators, the observed data were equal to or greater than the estimated data in the initial intervals. However, the HE percentage decreased starting in the 40–44-week interval (Figure 4). Furthermore, the difference between the estimated and observed data became statistically significant (*p* value < 0.0001) from the 50–54-week interval to the end of the productive period. Thus, the HE mean difference was unfavorable to the observed data, with 20.77% fewer hatched eggs than estimated (Figure 4).

**Figure 5:**
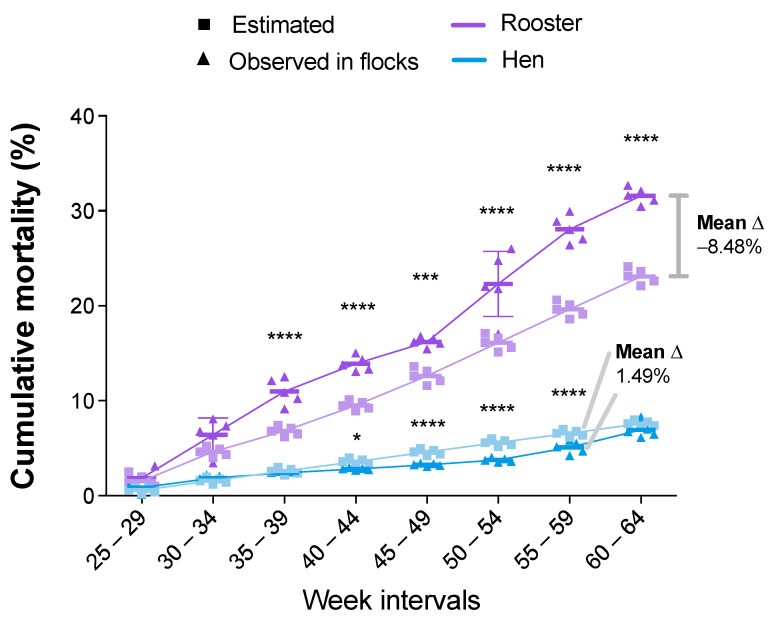
Percentages of cumulative rooster mortality (CRM) and cumulative hen mortality (CHM) for estimated and observed flocks. Results are presented as mean and standard deviation, and mean differences (shown as delta “Δ” mean) were calculated subtracting observed from estimated values. Comparisons were performed using the two-way ANOVA test followed by the Bonferroni correction post-hoc test. * *p* < 0.05; ** *p* < 0.005; *** *p* < 0.0005; **** *p* < 0.0001.

The following comparisons were made with respect to the mortality indicators. First, the cumulative hen mortality (CHM) showed equal or lower values in the data of the observed farms (Figure 5). Specifically, the difference was statistically significant from the 40–44-week interval to the 55–59-week interval. The last interval with statistical significance showed 1.49% fewer dead hens than the estimated. At the end of the productive period, the CHM mean difference was favorable to the observed data, with 0.66% fewer dead hens than estimated. In contrast, the cumulative rooster mortality (CRM), which counts the breeder males culled or dead, showed the same or greater rooster mortality in the observed data from the beginning of the productive period (Figure 5). Specifically, the difference was statistically significant (*p* value ≤ 0.0002) during the 35–39-week interval. At the end of the productive period, the CRM mean difference was unfavorable to the observed data, with 8.48% more dead roosters than estimated.

### 3.3. Histopathology and Electron Microscopy Findings

**Figure 6:**
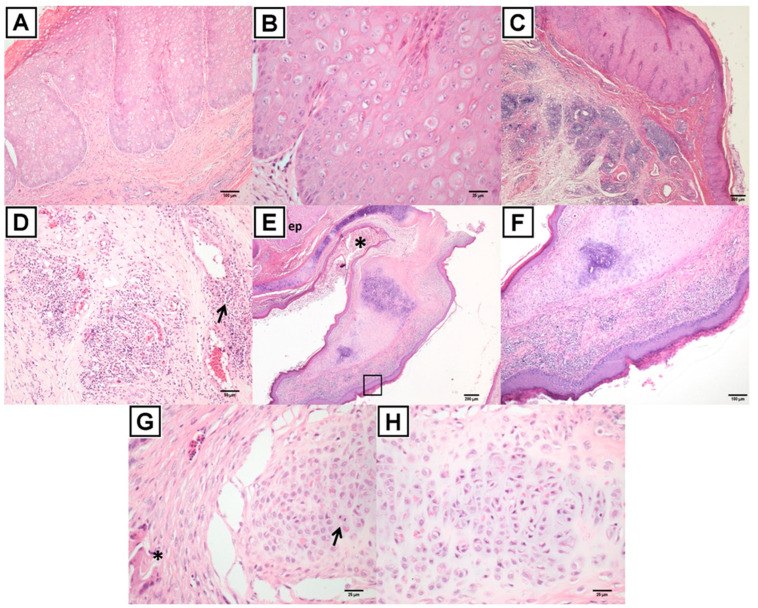
Photomicroscopy of the upper corneous beak and rostral nasal chamber. (**A**) Cornealized keratinized epithelium of the upper beak showing classic poxviral lesion, with irregular hyperplasia, ballooning, and intracytoplasmic inclusion corpuscle. (**B**) Zoomed-in hyperplastic epithelium with ballooned keratinocytes and intracytoplasmic inclusion corpuscle. (**C**) Transition region between epithelial proliferation with ballooning keratinocytes and intracytoplasmic inclusion corpuscle. Moderate perivascular inflammatory infiltrate in the lamina propria and nerve periphysis. (**D**) Zoomed-in C showing perivascular inflammatory infiltrate of lymphocytes, plasmocytes, and histiocytes. H&E staining. (**E**) Rostral nasal chamber shows hyperplasia of keratinocytes in the epithelium (ep) with Bollinger corpuscles. Lumen of the rostral nasal chamber with cell debris and erythrocytes (*). (**F**) Zoomed-in nasal septum with moderate inflammatory infiltrate in the lamina propria with exocytosis of heterophiles. Lymphoplasmacytic rhinitis. Notice an irregular cartilage. (**G**) Ventral region of the rostral nasal chamber and roof of the oral cavity where there are foci of infiltration of multinucleated giant cells (*) and choana cartilage with chondrocytes in mitosis (arrow), showing intracytoplasmic eosinophilic inclusion bodies (Bollinger corpuscles). (**H**) Nasal cartilage showing irregular chondrocytes and intracytoplasmic eosinophilic inclusion corpuscles (Bollinger corpuscles). H&E staining.

The keratinized epithelium of the upper and lower beak as well as the hard palate exhibited irregular hyperplasia with elongation and fusion of the epidermal cones with ballooning keratinocytes and intracytoplasmic eosinophilic inclusion bodies (Bollinger bodies) (Figure 6A–D, Figure S4A). The crust that covered part of the epithelium was characterized by fibrin heterophilic exudate with histiocytes, erythrocytes, and colonies of adhered bacteria. In some sections, hyphae had invaded the corneous beak, which are colored by the Schiff´s periodic acid (PAS) (Figure S4B). The basal lamina of the lining epithelium exhibited irregular hyperplasia, with frequent cells in mitosis and nuclear overlap. In the lamina propria, there was an inflammatory infiltrate of lymphoplasmacytic and perivascular histiocytes, peripheral nervous bundles to the interstitial space (Figure 6C,D), which was distributed among palatal salivary glands.

The lamina propria in the nasal septum and in the rostral nasal chamber (Figure 6E–H) presented a moderate interstitial inflammatory infiltrate composed of lymphocytes, plasmacytes, and heterophiles, which were sometimes in focal exocytosis. There were foci of multinucleated giant cells with inclusion bodies (Figure 6G) on the lamina propria between the epithelium of the hyperplasic palate and the ventral portion of the rostral nasal chamber. The cartilage of this site and from the sections of the rostral nasal chamber presented hyperplasia with chondrocytes in mitosis, structural disarrangements, and chondrocytes exhibiting intracytoplasmic eosinophilic inclusion bodies (Figure 6G–H).

In the lumen of the beak, there were cellular and nuclear remnants of inflammatory cells, epithelial cells, and erythrocytes (Figure 6E–F). Histopathological changes characterized stomatitis and ulcerative rhinitis, and crusted proliferative necrosis was determined from the presence of intracytoplasmic viral inclusion bodies (Bollinger bodies) in keratinocytes and chondrocytes.

**Figure 7:**
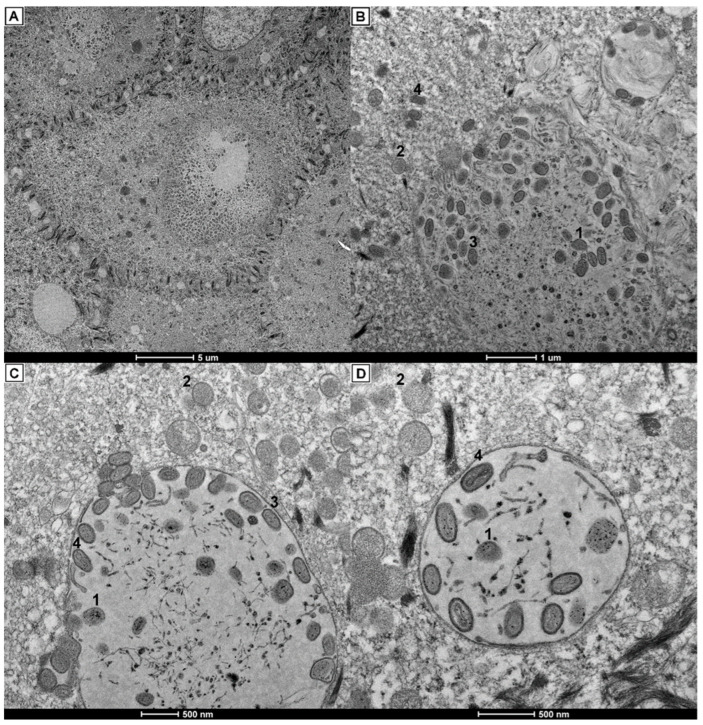
Electron micrographs showing poxvirus particles from the epithelial lesions in the beak. (**A**) Epithelial cells with medium to large intracytoplasmic inclusion bodies and vesicles containing granular material. (**B**) Viral particles in the periphery of the inclusion bodies containing all the poxvirus particle stages. (**C**) Mature viral particles at the periphery of the inclusion body membrane and protruding to the cytoplasm. Cytoplasmic viral particles with less dense nucleoids. (**D**) Enclosed inclusion body with incompletes, intermediates, and mature-form viral particles. Filaments and granules dispersed in the central part. Non-membrane-bound virus particles free in cytoplasm. Viral particles stages: incomplete form (1), spheric form (2), intermediate forms (3), and mature form (4).

In the transmission electron microscopy examination, epithelial cell membranes showed intracytoplasmic inclusions with viral particles in different stages (Figure 7A–D). Neighboring epithelial cells presented three types of inclusion bodies (Figure 7A): Type A inclusions (Bollinger bodies), which contained few or abundant viral particles with displacement of the cell nuclei; Type B inclusions, which had an electron-dense matrix of granules and fibrillar areas; and Type C inclusions, which were vesicles with small and peripheral granules. Bollinger bodies contained all the poxvirus particle stages (Figure 7B): the incomplete (with partial limiting membrane and fibrillar viroplasm), spherical (rounded to oval morphology with presence of double-layered membrane), intermediate (oval to elongated virions with dense to diffused nucleoid), and mature (with biconcave nucleoid and dumbbell-shaped) forms, which project toward the cytoplasm. Electron-dense filaments were found in the proximity of inclusion bodies (Figure 7C,D). Budding of the mature particles into the cytoplasm occurred individually or in groups, while some cytoplasmic viral particles could be observed with less dense nucleoids (Figure 7C). Few inclusion bodies contained all stages of poxvirus (Figure 7D).

### 3.4. Molecular Detection and Characterization of Avipoxvirus

**Figure 8:**
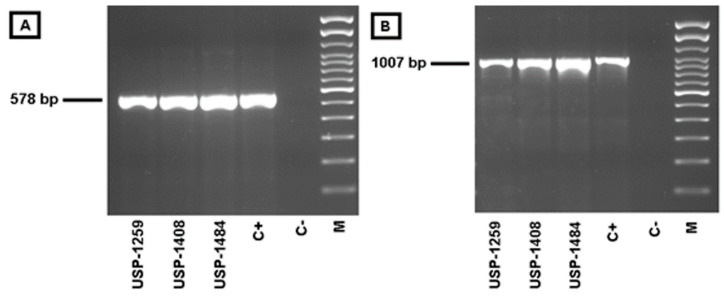
Electrophoresis for APV. (**A**) PCR for *P4b* APV gene. (**B**) PCR for *pol* APV gene. C+ = positive control, C− = negative control, M = molecular size marker (100 bp).

One sample from each outbreak (USP-1259, USP-1408, USP-1484) was tested for the presence of avipoxvirus. These samples consisted of a pool from injured pieces from the beaks of five affected roosters. PCR detection based on the major core protein amplified a fragment of approximately 580 base pairs (bp) in samples corresponding to the three outbreaks (Figure 8A). Similar results were found in the PCR assay for the DNA polymerase gene with amplified fragments of approximately 1007 bp (Figure 8B). In contrast, amplification of the LTR did not reveal the presence of reticuloendotheliosis virus in the field samples.

### 3.5. Sequencing and Phylogenetic Analysis

The obtained sequences were submitted to GenBank under the accession numbers MN257631 (*P4b* for USP-1259) and MN257632 (DNA *pol* for USP-1259); MW349699 (*P4b* for USP-1408) and MW349700 (DNA *pol* for USP-1408); and MW349701 (*P4b* for USP-1484) and MW349702 (DNA *pol* for USP-1484).

**Figure 9:**
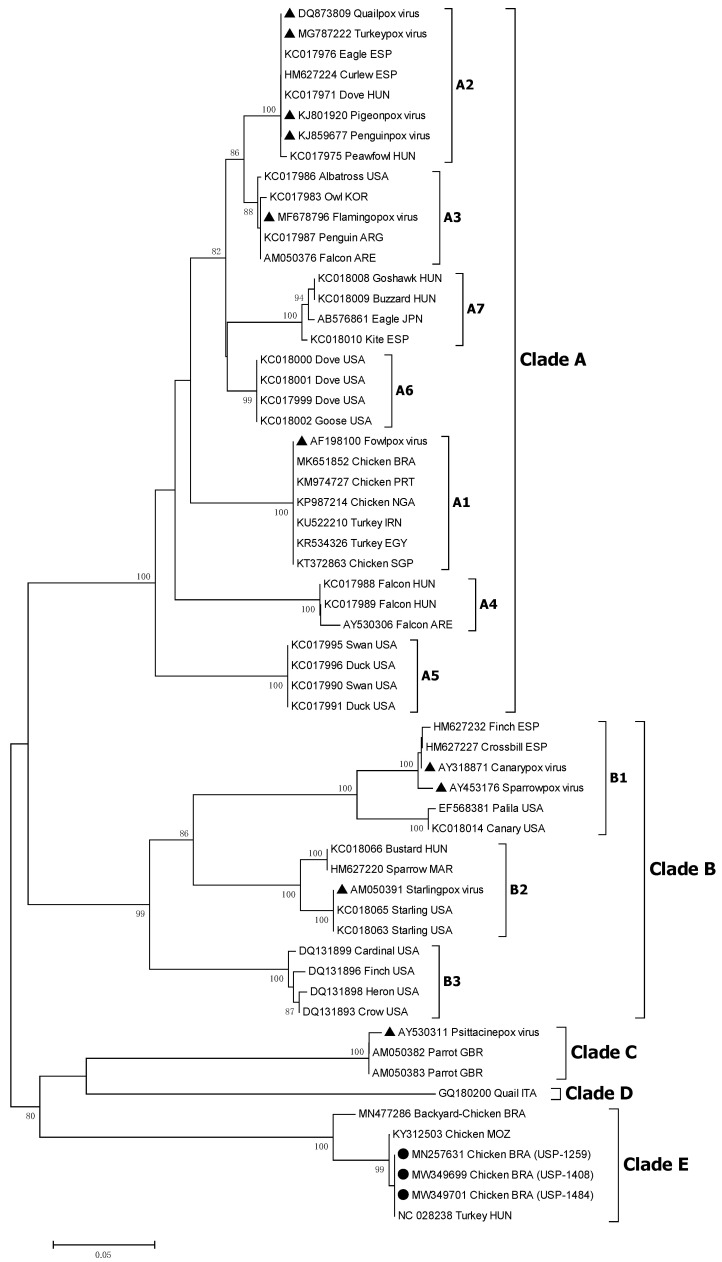
Phylogenetic analysis of partial major core (*P4b*) gene of APV strains. Strain names and GenBank accession numbers are shown. The black circles represent field APV strains of this study. Phylogenetic trees were constructed in MEGA v7.0 using the neighbor-joining method with 1000 bootstrap replicates. The evolutionary distances were computed using the Tamura 3-parameter model (T92 + I). Node values represent percentage support and scale bar represents the number of base substitutions per site. Brazilian strains of this study are represented by filled circles. Species representatives are shown with filled triangles.

**Figure 10:**
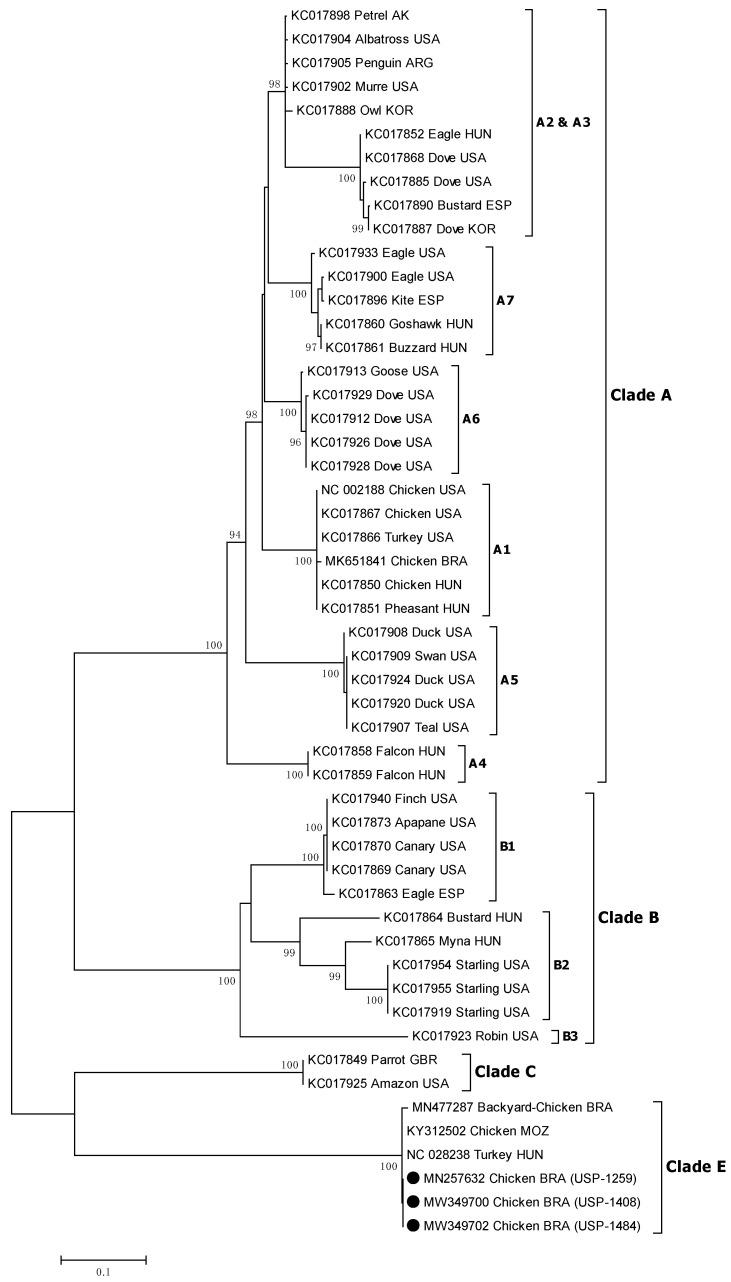
Phylogenetic analysis of partial DNA polymerase (*pol*) gene of APV strains. Strain names and GenBank accession numbers are shown. The black circles represent field APV strains of this study. Phylogenetic trees were constructed in MEGA v7.0 using the neighbor-joining method with 1000 bootstrap replicates. The evolutionary distances were computed using the Tamura-Nei model (TN93 + G + I). Node values represent percentage support and scale bar represents the number of base substitutions per site. Brazilian strains of this study are represented by filled circles.

Initial comparison revealed a total identity (100%) between all three analyzed outbreak samples, both in nucleotides and deduced amino acid sequences. The phylogenetic inference was based on the approaches widely used for avipoxvirus, which consist of sequencing and comparison of partial coding regions for proteins *P4b* core and DNA polymerase [13]. The samples corresponding to the three outbreaks were clustered together in the emerging clade E avipoxvirus, both for the major core protein and DNA polymerase genes, with 100% bootstrap support (Figure 9 and Figure 10). To date, all the available sequences corresponding to this clade (clade E) include the reference strain TKPV-HU1124/2011 (GenBank: KP728110) detected in turkeys from Hungary [17], isolates 608 and 980 detected in breeder chickens from Mozambique [18], and isolate APV08 detected in backyard chickens from South Brazil [19]. Additionally, FWPW was found in the subclade A1 as expected. In subclade A2, QUPV were found together with species PGPV, PNGPV, and TKPV. Subclade A3 included the species FLMPV. CNPV and SRPV were in subclade B1 while SLPV was in the subclade B2. Clade C contained the PSPV.

Sequence comparison of the Brazilian strains of the current study with other clade E avipoxvirus strains for partial *P4b* gene produced between 95.8 and 100.0% nucleotide identity (100.0% identity with the Hungarian strain, 99.7% identity with the Mozambique strain, and 95.8% identity with the backyard Brazilian strain) and between 92.5 and 100.0% identity in deduced amino acids (100.0% identity with the Hungarian and Mozambique strains, and 92.5% with the backyard Brazilian strain). With respect to the other clades, sequence identities ranged from 69.0 to 75.3% for nucleotides and 72.2 to 83.7% for deduced amino acids (Table 2).

Analysis of the partial *Pol* gene revealed 99.4 to 99.9% nucleotide identity with the other clade E avipoxvirus strains (99.9% identity with the Hungarian strain, 99.8% identity with the Mozambique strain, and 99.4% identity with the backyard Brazilian strain) and between 98.6 and 99.7% identity in deduced amino acids (99.7% identity with the Hungarian strain, 99.4% with the Mozambique strain, and 98.6% with the backyard Brazilian strain). Comparison against other clades showed nucleotide sequences ranging from 72.4 to 75.1% identity and from 74.8 to 79.3% identity for deduced amino acids (Table 2).

### 3.6. Development of qPCR Assays for the Detection and Quantification of Clade E Avipoxvirus

The standard curve for each evaluated qPCR assay demonstrated sufficient efficiency (>90%) (Table 3, Figure S5A–D), with values of 96.44%, 99.14%, and 96.49% (ORF49, 114, and 159 templates, respectively), and 95.03% for the beta-actin template. The correlation coefficient (R2) ranged from 0.998 to 0.999, and the standard error of the slope ranged from 0.016 to 0.029 (Figure S5A–D). The average melting temperature was 72.34 °C ± 0.14 for ORF49, 75.05 °C ± 0.15 for ORF114, 73.86 °C ± 0.15 for ORF159, and 84.10 °C ± 0.16 for the beta-actin gene assay.

The analytical sensitivity was evaluated through the limit of detection (LoD) and the limit of quantification (LoQ). LoD was determined in 1 GC per reaction, and LoQ was determined in 10 GC per reaction for each qPCR assay.

Subsequently, qPCR assays were used to test the field samples in triplicate. The results are summarized in Table 3. Quantification of avipoxvirus genome copies (GC) in each sample revealed high quantities of viral genomes with ranges of 0.39 × 10^6^ ± 0.02 to 2.51 × 10^6^ ± 0.65 (USP-1259), 0.77 × 10^5^ ± 0.15 to 4.38 × 10^5^ ± 1.41 (USP-1408), and 0.66 × 10^6^ ± 0.04 to 3.3 × 10^6^ ± 0.85 (USP-1484). In the case of beta-actin, the mean quantities of GC were 3.65 × 10^3^ ± 0.10 (USP-1259), 3.95 × 10^4^ ± 2.97 (USP-1408), and 1.64 × 10^5^ ± 0.27 (USP-1484). According to the results from each assay, the ratio of viral genome copies per cell was estimated to be in the ranges of 1.07–6.87 × 10^2^ (USP-1259), 0.19–1.11 × 10^1^ (USP-1408), and 1.22–6.08 × 10^1^ (USP-1484).

The specificity of the assays was tested in triplicate by using a dilution of 20 ng/µL fowlpox commercial vaccine (Poximune^®^ AE, Ceva Animal Health), as fowlpox was considered a close relative in terms of poultry infections. Indeed, the discrepancy analysis for designed primers revealed the highest identities’ percentages when comparing the clade E avipoxvirus with the fowlpox strains and/or the penguinpox strain (Figure S6, Table S5). The maximum identity percentages ranged from 35.71–55.56% (ORF49), 35.0–53.33% (ORF114), and 71.19–76.19% (ORF159) (Table S5). Thus, the fowlpox commercial vaccine (which tested positive in universal avipoxvirus PCR, Figure 8A,B) tested negative in all 3 qPCR assays specific for clade E avipoxvirus.

## 4. Discussion

During recent decades, Brazil has emerged alongside the U.S. and China as one of the most important countries for the poultry industry, both in terms of production and exportation. Thus, the emergence of new variant pathogens, especially those presenting immune evasive characteristics, is of high concern. In the present study, we detected outbreaks caused by the clade E avipoxvirus for the first time in Brazilian commercial poultry flocks. These outbreaks exhibited particularly exacerbated clinical signs in the beak, increased mortality in roosters, and impaired hatchability and egg fertility due to weakness and pain in the beak during the mating.

The natural course of mild cutaneous infections in chickens persists three to four weeks. This period is often extended in complicated outbreaks [1]. In the present study, clinical signs were observed for up to 35 weeks. This extended duration could be associated with coinfection infections [35], virulence of the strains detected [1,36], or the persistence of contamination sources or vectors such as insects [37].

Gross examination of the affected birds indicated possible poxvirus infection with proliferative lesions across the head. Some crusts and scabs were distributed in the combs, wattles, and eyelids of the birds, which were similar to the classic cutaneous form of the infection. Interestingly, moderate to severe necrotic lesions were focused on the nostrils and beaks and were most frequently found in roosters. These lesions were deleterious, causing an increased rooster mortality (8.48%). Although the classic lesions of the fowlpox virus can manifest in any cutaneous skin, a higher frequency of fatal cases has been seen with lesions in the eyelids, beaks, and mouth commissures in chickens [18,35], crows [38], and psittacines [39,40]. These lesions limit vision and feeding ability [1].

We performed descriptive statistical inferences to evaluate the differential disease process in terms of productive and survival flock parameters. We analyzed the egg production-related parameters HDEP and CEHP. Both demonstrated a normal or above normal productive performance. Although the egg production index began to decrease when the pox lesions emerged in the flock, it seems fortuitous. This decrease may be a normal consequence of the performance decay associated with the age of the breeder hens [41,42]. In fact, it is possible that the difference in favor of the observed flocks would have been statistically significant, ruling out possible detrimental consequences on HDPE. Based on the good HDEP and CEHP performances, we inferred that the breeder hens of the studied flocks were physically and physiologically unimpaired in terms of egg laying.

In counterpart, a remarkable drop and undesirable effect were observed in the progression of the hatched egg count. The observed HE curve intersected the estimated HE curve between the 35–39-week interval and the 40–44-week interval. As the initial pox lesion detection occurred at week 30 in roosters, the most likely scenario to explain this pattern was a negative mid-term impact in fertility performance suffered by the roosters because of the progressive lesions. This effect increased over the productive time and suggested a relationship with the persistence and progression of the lesions. Fowlpox infections can cause drop in egg production because of the intensity of the proliferative lesions [43] and the consequent impaired fertility [1]. Controversially, in this study we found impaired hatchability (20.77% lower than estimated) but without a drop in egg production.

Analyses of mortality found sex differences. Breeder hens presented normal or healthier CHM progression. In contrast, in breeder roosters, the observed CRM was higher than the estimated. This difference between groups became statistically significant after the 35–39-week interval. This reflects the negative mid-term impact on the roosters after the detection of the disease at week 30.

The analysis of the results altogether shows that only the rooster breeders were significatively affected (by increased mortality) and this impacted the hatchability indices directly. Sex differences in clinical symptomatology could be influenced by physiological, anatomical, mechanical, or immunological factors [44]. Additionally, Barbour et al. [45] reported sex differences in immune responses to fowlpox vaccination. This event could have happened on the farms studied. However, vaccination monitoring revealed successful “takes” in 98% of the birds (including roosters and hens). In addition, it has been reported that the percentages of “takes” are proportional to antibody titers [46]. In the opposite way, the morbidity values were significant (45% of the roosters and 20% of the hens). Considering that, we suggest a sex difference in susceptibility and severity to clade E avipoxvirus infection and disease. Consequently, severe impairment of the rooster’s beak anatomy, progressive lesions affecting reproduction, and starvation causing the increased mortality.

Histopathology analyses were performed in sections of nodules, affected nostrils, beak, and palate. The findings in the lining and keratinized epithelium included inflammation, hyperplasia, ballooning, and the presence of Bollinger bodies. These findings are common in avipoxvirus infections [1,47]. Remarkably, we also found affected connective tissue, with hyperplasia and inclusion bodies in chondrocytes. A similar phenomenon was previously reported in captive peafowl [48]. However, that study did not identify the poxvirus genotype. A multiple-tissue poxvirus infection is suggested to be associated with the exacerbated signs caused by this novel species. In these outbreaks, we also identified secondary infections. Secondary bacterial and fungal infections can also be seen in avipoxvirus lesions and are associated with pre-existing immunosuppression [49].

Electron micrographs of epithelial cells revealed poxvirus infection, with intracytoplasmic inclusions showing viral particles in different stages of formation (incomplete, spherical, intermediate, and mature forms). It was also possible to see dense filaments and the budding process of the mature viral particles. This evidence is in accordance with typical poxvirus infections [50,51].

We initially contemplated the immunosuppression and exacerbated clinical manifestations as potentially being due to coinfection with reticuloendotheliosis virus, as previously reported [9,10,47,52]. This hypothesis was rejected, as the REV genome was not detected. However, immunosuppression caused by stress, mycotoxins, or coinfection with other immunosuppressor viruses (IS) affecting these farms cannot be totally discarded. IS can increase susceptibility to other pathogens and interfere with acquired vaccinal immunization [53,54]. Additionally, it was reported that the coinfection of avipoxvirus with bacteria, fungus, or other viruses can alter and exacerbate the pathology and cause an increase in mortality [55,56].

Phylogenetic analyses of the *P4b* and *Pol* genes both demonstrated a complete supported clustering with the clade E avipoxvirus. In addition, identity sequence comparison confirmed the inclusion of our studied strains in clade E, with 95.8 to 100% identity against the other avipoxvirus strains of this clade for the *P4b* gene and 99.4 to 99.9% for the *pol* gene. In contrast, this identity was lower when compared with other avipoxvirus clades, ranging from 69.0 to 75.3% and 72.2 to 75.0% for the *P4b* and *Pol* genes, respectively. As previously mentioned, the other clade E strains were detected in only 3 studies: in turkeys from Hungary [17], in breeder chickens from Mozambique [18], and in backyard chickens from Brazil [19]. Interestingly, the most identical sequences to our studied strains corresponded to the Hungarian clade E prototype from turkeys, both in *P4b* and *pol* genes, and the most distant was the Brazilian backyard chicken strain. This is noteworthy because it might indicate the geographical origin of our strains. The hypothesis of a possible route associated with the commercial avian industry cannot be discarded.

Despite avipoxvirus causing disease in a broad range of birds [6], each specific genotype is mainly adapted to a specific host family or order, especially in wild birds [57,58]. However, there are exceptions to this rule [7]. Fowlpox-like viruses, canarypox-like viruses, and psittacinepox viruses have been proposed to have broader ranges of hosts [1]. This pattern could be influenced by the ecological niche, habitat, and geography of the host species [13]. The broader the host range is, the greater the risk of emergent outbreaks; the emergence of new outbreaks is directly related to the number of susceptible hosts [13]. In our study, we report that clade E avipoxvirus, which was originally reported to affect turkeys, can also cause disease in chickens.

Analysis of the complete genome from the clade E prototype (TKPV-HU1124/2011) revealed an intriguing question. The reduction in the number of genes due to evolution or host–pathogen adaptation could impact the virulence and severity of the virus. However, the clade E avipoxvirus has a compact genome (188 kbp) and expresses similar or even worse clinical manifestations [17,18]. This was also observed in our current study as increased severity in roosters and reduced hatchability performance. Furthermore, it has been proposed that the quantity of ankyrin repeat genes may be associated with a narrowing of the host range [1]; however, the clade E avipoxvirus genome has 16 copies of these genes, whereas the fowlpox virus genome has 31 copies [28], which appears to contradict this proposal. Moreover, the clade E avipoxvirus was situated in a basal position in the avipoxvirus phylogeny and was suggested to represent an ancient evolutionary lineage [17]. This could also permit a broader host range. Further studies are necessary to confirm this likelihood, which is of high priority because of the high potential risk to commercial poultry and wild avifauna.

To quantify and detect this emerging avipoxvirus, we developed three quantitative PCR assays based on the prototype clade E reference genome (KP728110). Our assay analyses showed good results. The efficiency of the assays was 96.44 to 99.14%. The sensitivity had a limit of detection of one genome copy per reaction and a limit of quantification of ten genome copies per reaction. When tested against the fowlpox virus strain, there was no cross reaction, indicating high specificity. Then, we used qPCR assays to detect and quantify the relative viral charge against a chicken housekeeping gene (beta-actin). Our results revealed that the viral genome copy numbers ranged from 1.9 to 687 per cell in the samples of the studied outbreaks.

One of the most alarming aspects of this outbreak was the exacerbated clinical manifestation despite the double-vaccinated status of the affected birds. This is of great concern in commercial poultry farming. Outbreaks of fowlpox have been reported in vaccinated birds in recent decades [59,60]. In some cases, these are due to the integration of the reticuloendotheliosis virus [9,11]. However, in previous reports of clade E avipoxvirus infections in birds vaccinated against fowlpox virus, these outbreaks may possibly be associated with the presence of this variant avipoxvirus [17,18]. Genomic and antigenic heterogeneity in avipoxviruses may contribute to the differences in elicited immune responses, impacting the level of protection [61,62].

Our study reveals the emergence and spread of this emerging pathogen associated with harmful outbreaks and exacerbated clinical manifestations in commercial poultry farms. This study also highlights the economic significance of this pathogen for poultry farming and suggests a reassessment of the current vaccination programs.

## 5. Conclusions

Avipoxvirus infections are constantly reported in multiple avian hosts. In some of these cases, the presence of a new genotype or species is detected. The emergence of new virus can trigger a series of damages in poultry farming and in the environment. Thus, this study describes outbreaks of a recently reported clade E avipoxvirus in Brazilian breeder flocks vaccinated against fowlpox virus. Among the detrimental consequences were reduced laying performance, exacerbated beak tropism, and increased rooster mortality. We also provided qPCR assays for differential detection and quantification of this virus. An opportune detection and appropriate identification of this virus must help the preventive and corrective measures to avoid future outbreaks. Additional studies must be carried out to understand the immune status of chickens vaccinated with fowlpox virus followed by an experimental challenge with the clade E avipoxvirus.

## Figures and Tables

**Table 1:** Detailed list of primers used in the present study.

Target Gene	Primer	Sequence (5’ > 3’)	Location *	Product Length
ORF49	qEPox49-F	GCTGATTACGGGATCTATGTTG	52,912–52,933 ^A^	107 bp
	qEPox49-R	TAGTTGCCTTTGTATCTGCG	52,999–53,018 ^A^	
	E49-Fex	CTGCCAATGCTATCGATACC	52,730–52,749 ^A^	449 bp
	E49-Rex	GCAACAAAACGAGAGGTTTC	53,159–53,178 ^A^	
ORF114	qEPox114-F	GGACTCAACAAACGTGCTAT	118,713–118,732 ^A^	177 bp
	qEPox114-R	CTGTTCATTAGACGTCGTGC	118,870–118,889 ^A^	
	E114-Fex	GGCTAGATTGATAACAGCTATGG	118,494–118,516 ^A^	434 bp
	E114-Rex	TGATAGTCGTCTTTATACGAGCAC	118,904–118,927 ^A^	
ORF159	qEPox159-F	AGATGGTGATGATTACGGATG	168,643–168,663 ^A^	104 bp
	qEPox159-R	CAGATACGCTAGACCAATCAG	168,726–168,746 ^A^	
	E159-Fex	CGTATATGCCTCTACTTGTAATTG	168,451–168,474 ^A^	511 bp
	E159-Rex	ACTTTCTCCCTTATCAGCAAC	168,941–168,961 ^A^	
B actin	qβactin-F	TCAGACTCTGGAGTGCCTTG	3913–3932 ^B^	101 bp
	qβactin-R	GGTCAGATGCAGTGTGATGG	3994–4013 ^B^	
	βactin-Fex	GCTGCTCTTAGCACACCTCTT	3694–3714 ^B^	346 bp
	βactin-Rex	GCAATGATCTGCAGGAGAGA	4020–4039 ^B^	

* According to reference genomes for ^A^: TKPV (GenBank: KP728110), ^B^: *Gallus gallus* beta-actin (GenBank: X00182).

**Table 2:** Nucleotide and amino acid identity of studied strains compared with other APVs.

CLADE	SUBCLADE	Comparative Identity with Strains from This Study			
		Partial *P4b* Gene		Partial *Pol* Gene	
		Nucleotides	Amino Acids	Nucleotides	Amino Acids
A	A1	72.5–73.9	75.9–76.6	72.3–73.6	77.4–78.1
	A2	73.2–74.9	75.6–80.3	72.7–74.3	75.7–77.0
	A3	72.7–74.1	75.4–76.8	73.8–74.2	76.4–77.2
	A4	72.2–73.5	74.6–75.1	74.6–74.7	77.7–78.2
	A5	73.3–73.5	74.5–74.6	72.5–72.6	76.4–76.8
	A6	72.8–73.0	75.7–75.8	74.3–75.0	78.3–79.3
	A7	72.0–73.6	75.0–77-8	72.4–74.5	77.1–78.2
B	B1	71.1–71.9	73.9–74.3	73.8–74.0	76.9–77.4
	B2	74.6–75.3	76.3–77.3	72.2–74.3	74.8–77.2
	B3	72.8–73.4	76.6–77.2	74.0	77.2–77.4
C		74.5–75.2	82.7–83.7	73.3–73.4	78.7–79.3
D		69.0	76.9	–	–
E		95.8–100.0	92.5–100.0	99.4–99.9	98.6–99.7

**Table 3:** Results of qPCR assays in studied outbreaks.

Target	Standard Curve		USP-1259		USP-1408		USP-1484	
	E ^A^	Tm	GC	GC/Cell	GC	GC/Cell	GC	GC/Cell
ORF49	96.44%	72.34 °C ± 0.14	3.95 × 10^5^ ± 0.24	1.07 × 10^2^	7.66 × 10^4^ ± 1.49	1.94 × 10^0^	9.83 × 10^5^ ± 2.11	1.81 × 10^1^
ORF114	99.14%	75.05 °C ± 0.15	2.51 × 10^6^ ± 0.65	6.87 × 10^2^	4.38 × 10^5^ ± 1.41	1.11 × 10^1^	3.30 × 10^6^ ± 0.85	6.08 × 10^1^
ORF159	96.49%	73.86 °C ± 0.15	4.00 × 10^5^ ± 0.08	1.09 × 10^2^	1.08 × 10^5^ ± 0.04	2.73 × 10^0^	6.63 × 10^5^ ± 0.35	1.22 × 10^1^
B-actin	95.03%	84.10 °C ± 0.16	3.65 × 10^3^ ± 0.10	─	3.95 × 10^4^ ± 2.97	─	1.64 × 10^5^ ± 0.27	─
GC Range			1.07–6.87 × 10^2^ GC/cell		0.19–1.11 × 10^1^ GC/cell		1.22–6.08 × 10^1^ GC/cell	

^A^ Efficiency.

## Data Availability

The obtained sequences were submitted to GenBank under the accession numbers MN257631 (*P4b* for USP-1259) and MN257632 (*DNA pol* for USP-1259); MW349699 (*P4b* for USP-1408) and MW349700 (*DNA pol* for USP-1408); and MW349701 (*P4b* for USP-1484) and MW349702 (*DNA pol* for USP-1484).

## Supplementary Materials

The following supporting information can be downloaded at: https://www.mdpi.com/article/10.3390/v14040773/s1, Table S1. Zootechnical performance data of Flock USP-1408; Table S2. Zootechnical performance data of Flock USP-1484; Table S3. Weighted Weekly Egg Breeding Performance and Mortality of Flocks (USP-1408 and USP-1484); Table S4. Avipoxvirus strains with available complete genome selected for sequence comparison; Table S5. Discrepancies analysis of primers for specific detection of clade E avipoxvirus; Figure S1. Electrophoresis for external primers products; Figure S2. Clinical signs observed in roosters and hens of broiler breeders by different degrees of severity; Figure S3. Beak lesions of broiler breeders varying from erosions in nostrils to profound damage of choanas; Figure S4. Photomicroscopy of irregular epithelium of the upper beak; Figure S5. Standard curve plots for qPCR assays developed for detection and quantification of Clade E Avipoxvirus. Figure S6. Target regions of primers.

## References

[1] Tripathy D.N., Reed W.M.P. (2020). Pox. Diseases of Poultry.

[2] Walker P.J., Siddell S.G., Lefkowitz E.J., Mushegian A.R., Adriaenssens E.M., Dempsey D.M., Dutilh B.E., Harrach B., Harrison R.L., Hendrickson R.C. (2020). Changes to Virus Taxonomy and the Statutes Ratified by the International Committee on Taxonomy of Viruses. Arch. Virol..

[3] Kim T.-J., Schnitzlein W.M., McAloose D., Pessier A.P., Tripathy D.N. (2003). Characterization of an Avianpox Virus Isolated from an Andean Condor (*Vultur gryphus*). Vet. Microbiol..

[4] Sarker S., Das S., Lavers J.L., Hutton I., Helbig K., Imbery J., Upton C., Raidal S.R. (2017). Genomic Characterization of Two Novel Pathogenic Avipoxviruses Isolated from Pacific Shearwaters (*Ardenna* spp.). BMC Genom..

[5] Sarker S., Batinovic S., Talukder S., Das S., Park F., Petrovski S., Forwood J.K., Helbig K.J., Raidal S.R. (2020). Molecular Characterisation of a Novel Pathogenic Avipoxvirus from the Australian Magpie (*Gymnorhina tibicen*). Virology.

[6] Bolte A.L., Meurer J., Kaleta E.F. (1999). Avian Host Spectrum of Avipoxviruses. Avian Pathol..

[7] Giddens W.E., Swango L.J., Henderson J.D., Lewis R.A., Farner D.S., Carlos A., Dolowy W.C. (1971). Canary Pox in Sparrows and Canaries (Fringillidae and in Weavers (Ploceidae). Pathology and Host Specificity of the Virus. Vet. Pathol..

[8] Giotis E.S., Skinner M.A. (2019). Spotlight on Avian Pathology: Fowlpox Virus. Avian Pathol..

[9] Chacón R.D., Astolfi-Ferreira C.S., De la Torre D.I., de Sá L.R.M., Piantino Ferreira A.J. (2020). An Atypical Clinicopathological Manifestation of Fowlpox Virus Associated with Reticuloendotheliosis Virus in Commercial Laying Hen Flocks in Brazil. Transbound. Emerg. Dis..

[10] Joshi L.R., Bauermann F.V., Hain K.S., Kutish G.F., Armién A.G., Lehman C.P., Neiger R., Afonso C.L., Tripathy D.N., Diel D.G. (2019). Detection of Fowlpox Virus Carrying Distinct Genome Segments of Reticuloendotheliosis Virus. Virus Res..

[11] Singh P., Schnitzlein W.M., Tripathy D.N. (2003). Reticuloendotheliosis Virus Sequences within the Genomes of Field Strains of Fowlpox Virus Display Variability. J. Virol..

[12] Srinivasan V., Tripathy D.N. (2005). The DNA Repair Enzyme, CPD-Photolyase Restores the Infectivity of UV-Damaged Fowlpox Virus Isolated from Infected Scabs of Chickens. Vet. Microbiol..

[13] Gyuranecz M., Foster J.T., Dán Á., Ip H.S., Egstad K.F., Parker P.G., Higashiguchi J.M., Skinner M.A., Höfle U., Kreizinger Z. (2013). Worldwide Phylogenetic Relationship of Avian Poxviruses. J. Virol..

[14] Lawson B., Lachish S., Colvile K.M., Durrant C., Peck K.M., Toms M.P., Sheldon B.C., Cunningham A.A. (2012). Emergence of a Novel Avian Pox Disease in British Tit Species. PLoS ONE.

[15] MacDonald A.M., Gibson D.J., Barta J.R., Poulson R., Brown J.D., Allison A.B., Nemeth N.M. (2019). Bayesian Phylogenetic Analysis of Avipoxviruses from North American Wild Birds Demonstrates New Insights into Host Specificity and Interspecies Transmission. Avian Dis..

[16] Manarolla G., Pisoni G., Sironi G., Rampin T. (2010). Molecular Biological Characterization of Avian Poxvirus Strains Isolated from Different Avian Species. Vet. Microbiol..

[17] Bányai K., Palya V., Dénes B., Glávits R., Ivanics É., Horváth B., Farkas S.L., Marton S., Bálint Á., Gyuranecz M. (2015). Unique Genomic Organization of a Novel Avipoxvirus Detected in Turkey (*Meleagris gallopavo*). Infect. Genet. Evol..

[18] Mapaco L.P., Lacerda Z., Monjane I.V.A., Gelaye E., Sussuro A.H., Viljoen G.J., Dundon W.G., Achá S.J. (2017). Identification of Clade E Avipoxvirus, Mozambique, 2016. Emerg. Infect. Dis..

[19] Ribeiro L.C., Monteiro F.L., Chagas D.B., D’Ávila Vargas G., de Lima M., Fischer G., de Oliveira Hübner S. (2020). Identification of Clade E Avipoxvirus in Brazil. Avian Dis..

[20] Watanabe I., Yamada E. (1983). The Fine Structure of Lamellated Nerve Endings Found in the Rat Gingiva. Arch. Histol. Jpn..

[21] Ciena A.P., de Almeida S.R.Y., Alves P.H.d.M., Bolina-Matos R.d.S., Dias F.J., Issa J.P.M., Iyomasa M.M., Watanabe I. (2011). Histochemical and Ultrastructural Changes of Sternomastoid Muscle in Aged Wistar Rats. Micron.

[22] Spurr A.R. (1969). A Low-Viscosity Epoxy Resin Embedding Medium for Electron Microscopy. J. Ultrastruct. Res..

[23] Ciena A.P., de Sousa Bolina C., de Almeida S.R.Y., Rici R.E.G., de Oliveira M.F., da Silva M.C.P., Miglino M.A., Watanabe I. (2013). Structural and Ultrastructural Features of the Agouti Tongue (*Dasyprocta aguti linnaeus*, 1766). J. Anat..

[24] Huw Lee L., Hwa Lee K. (1997). Application of the Polymerase Chain Reaction for the Diagnosis of Fowl Poxvirus Infection. J. Virol. Methods.

[25] Cao W., Mays J., Dunn J., Fulton R., Silva R., Fadly A. (2013). Use of Polymerase Chain Reaction in Detection of Marek’s Disease and Reticuloendotheliosis Viruses in Formalin-Fixed, Paraffin-Embedded Tumorous Tissues. Avian Dis..

[26] Katoh K., Standley D.M. (2013). MAFFT Multiple Sequence Alignment Software Version 7: Improvements in Performance and Usability. Mol. Biol. Evol..

[27] Kumar S., Stecher G., Tamura K. (2016). MEGA7: Molecular Evolutionary Genetics Analysis Version 7.0 for Bigger Datasets. Mol. Biol. Evol..

[28] Afonso C.L., Tulman E.R., Lu Z., Zsak L., Kutish G.F., Rock D.L. (2000). The Genome of Fowlpox Virus. J. Virol..

[29] Laidlaw S.M., Skinner M.A. (2004). Comparison of the Genome Sequence of FP9, an Attenuated, Tissue Culture-Adapted European Strain of Fowlpox Virus, with Those of Virulent American and European Viruses. J. Gen. Virol..

[30] Offerman K., Carulei O., van der Walt A.P., Douglass N., Williamson A.-L. (2014). The Complete Genome Sequences of Poxviruses Isolated from a Penguin and a Pigeon in South Africa and Comparison to Other Sequenced Avipoxviruses. BMC Genom..

[31] Carulei O., Douglass N., Williamson A.-L. (2017). Comparative Analysis of Avian Poxvirus Genomes, Including a Novel Poxvirus from Lesser Flamingos (*Phoenicopterus minor*), Highlights the Lack of Conservation of the Central Region. BMC Genom..

[32] Croville G., Le Loc’h G., Zanchetta C., Manno M., Camus-Bouclainville C., Klopp C., Delverdier M., Lucas M.-N., Donnadieu C., Delpont M. (2018). Rapid Whole-Genome Based Typing and Surveillance of Avipoxviruses Using Nanopore Sequencing. J. Virol. Methods.

[33] Kost T.A., Theodorakis N., Hughes S.H. (1983). The Nucleotide Sequence of the Chick Cytoplasmic Beta-Actin Gene. Nucleic Acids Res..

[34] Chacón R.D., Astolfi-Ferreira C.S., Chacón J.L., Nuñez L.F.N., De la Torre D.I., Piantino Ferreira A.J. (2019). A Seminested RT-PCR for Molecular Genotyping of the Brazilian BR-I Infectious Bronchitis Virus Strain (GI-11). Mol. Cell. Probes.

[35] Karpińska T.A., Kozaczyński W., Niemczuk K., Jasik A., Kycko A., Reichert M. (2014). Mixed Infection by Fowlpox Virus and Chlamydophila Psittaci in a Commercial Laying Hen Flock. Acta Vet. Hung..

[36] Zhao K., He W., Xie S., Song D., Lu H., Pan W., Zhou P., Liu W., Lu R., Zhou J. (2014). Highly Pathogenic Fowlpox Virus in Cutaneously Infected Chickens, China. Emerg. Infect. Dis..

[37] Yeo G., Wang Y., Chong S.M., Humaidi M., Lim X.F., Mailepessov D., Chan S., How C.B., Lin Y.N., Huangfu T. (2019). Characterization of Fowlpox Virus in Chickens and Bird-Biting Mosquitoes: A Molecular Approach to Investigating Avipoxvirus Transmission. J. Gen. Virol..

[38] Fukui D., Nakamura M., Yamaguchi T., Takenaka M., Murakami M., Yanai T., Fukushi H., Yanagida K., Bando G., Matsuno K. (2016). An Epizootic of Emerging Novel Avian Pox in Carrion Crows (*Corvus corone*) and Large-Billed Crows (*Corvus macrorhynchos*) in Japan. J. Wildl. Dis..

[39] González-Hein G., González C., Hidalgo H. (2008). Case Report: An Avian Pox Outbreak in Captive Psittacine Birds in Chile. J. Exot. Pet Med..

[40] Murer L., Westenhofen M., Kommers G.D., Furian T.Q., Borges K.A., Kunert-Filho H.C., Streck A.F., Lovato M. (2018). Identification and Phylogenetic Analysis of Clade C Avipoxvirus in a Fowlpox Outbreak in Exotic Psittacines in Southern Brazil. J. Vet. Diagn. Investig..

[41] Robinson F.E., Hardin R.T., Robblee A.R. (1990). Reproductive Senescence in Domestic Fowl: Effects on Egg Production, Sequence Length and Inter-Sequence Pause Length. Br. Poult. Sci..

[42] Zakaria A.H., Omar O.H. (2013). Egg Laying Pattern, Egg Weight, Body Weight at Hatch, and Sex Ratio Bias Relative to Oviposition Time of Young-and Mid-Age Broiler Breeders. Anim. Reprod. Sci..

[43] Hassan M.S.H., Abdul-Careem M.F. (2020). Avian Viruses That Impact Table Egg Production. Animals.

[44] Taylor R.L., Cotter P.F., Wing T.L., Briles W.E. (1987). Major Histocompatibility (B) Complex and Sex Effects on the Phytohaemagglutinin Wattle Response. Anim. Genet..

[45] Barbour E.K., Hamadeh S.K., Hilan C., Abbas S.S. (1995). Comparison of Immunity and Resistance to Diseases in Male and Female Poultry Breeders in Lebanon. Trop. Anim. Health Prod..

[46] Barreda C.B. (2016). Relationship Between Values of Fowlpox ELISA and the Presence of “Takes” After Vaccination. Avian Dis..

[47] Weli S.C., Tryland M. (2011). Avipoxviruses: Infection Biology and Their Use as Vaccine Vectors. Virol. J..

[48] Khan A., Yousaf A., Khan M.Z., Siddique M., Gul S.T., Mahmood F. (2009). Cutaneous Form of Pox Infection among Captive Peafowl (*Pavo cristatus*) Chicks. Avian Pathol..

[49] Tripathy D.N., Schnitzlein W.M., Morris P.J., Janssen D.L., Zuba J.K., Massey G., Atkinson C.T. (2000). Characterization of Poxviruses from Forest Birds in Hawaii. J. Wildl. Dis..

[50] Groupe V., Oskay J., Rake G. (1946). Electron Micrographs of the Elementary Bodies of Fowl Pox and Canary Pox. Proc. Soc. Exp. Biol. Med..

[51] Tudor D.C., Rue J.W., Woodward H.L. (1975). Electron Scanning Microscope Studies in Pigeon Pox Virus. Poult. Sci..

[52] Chacón R.D., Astolfi-Ferreira C.S., Guimarães M.B., Torres L.N., De la Torre D.I., de Sá L.R.M., Piantino Ferreira A.J. (2019). Detection and Molecular Characterization of a Natural Coinfection of Marek’s Disease Virus and Reticuloendotheliosis Virus in Brazilian Backyard Chicken Flock. Vet. Sci..

[53] Hoerr F.J. (2010). Clinical Aspects of Immunosuppression in Poultry. Avian Dis..

[54] Gimeno I.M., Schat K.A. (2018). Virus-Induced Immunosuppression in Chickens. Avian Dis..

[55] Ogasawara F., Yamamoto Y., Sato Y., Fukunari K., Murata K.-I., Yaegashi G., Goto M., Murakami R. (2016). Concurrent Fowlpox and Candidiasis Diseases in Backyard Chickens with Unusual Pox Lesions in the Bursa of Fabricius. Avian Dis..

[56] Shivaprasad H.L., Kim T., Tripathy D., Woolcock P.R., Uzal F. (2009). Unusual Pathology of Canary Poxvirus Infection Associated with High Mortality in Young and Adult Breeder Canaries (*Serinus canaria*). Avian Pathol..

[57] Landolt M., Kocan R.M. (1976). Transmission of Avian Pox from Starlings to Rothchild’s Mynahs. J. Wildl. Dis..

[58] Donnelly T.M., Crane L.A. (1984). An Epornitic of Avian Pox in a Research Aviary. Avian Dis..

[59] Fatunmbi O.O., Reed W.M. (1996). Evaluation of a Commercial Modified Live Virus Fowl Pox Vaccine for the Control of “Variant” Fowl Poxvirus Infections. Avian Dis..

[60] Fallavena L.C.B., Canal C.W., Salle C.T.P., Moraes H.L.S., Rocha S.L.S., Pereira R.A., da Silva A.B. (2002). Presence of Avipoxvirus DNA in Avian Dermal Squamous Cell Carcinoma. Avian Pathol..

[61] Schnitzlein W.M., Ghildyal N., Tripathy D.N. (1988). Genomic and Antigenic Characterization of Avipoxviruses. Virus Res..

[62] Singh P., Kim T.J., Tripathy D.N. (2000). Re-Emerging Fowlpox: Evaluation of Isolates from Vaccinated Flocks. Avian Pathol..

