# Supplementary material for: Outbreaks of Avipoxvirus Clade E in Vaccinated Broiler Breeders with Exacerbated Beak Injuries and Sex Differences in Severity
Source: Viruses. 2022 Apr 8;14(4):773. doi: 10.3390/v14040773 (PMC9028998; doi:10.3390/v14040773)
Supplement: Supplementary file 1 [file viruses-14-00773-s001.zip › viruses-1615006-supplementary.pdf]

viruses

MDPI

Supplementary data

Outbreaks of avipoxvirus clade E in vaccinated broiler breeders with exacerbated beak injuries and sex differences in severity

Ruy D. Chacón[1,2], Claudete S. Astolfi-Ferreira[1], Patrícia C. Pereira[3], Mario S. Assayag Jr[1], Antony B. Campos-Salazar[4], David De la Torre[1,5], Lilian R. M. de Sá[1], Sônia R. Yokomizo[6], Rose Elí Grassi Rici[7], and Antonio J. Piantino Ferreira[1,*]

[1] Department of Pathology, School of Veterinary Medicine, University of São Paulo, Av. Prof. Orlando M. Paiva, 87, 05508-270, São Paulo, Brazil; ruychaconv@usp.br (R.D.C.); csastolfi@gmail.com (C.S.A.-F.); sergioassayag@uol.com.br (M.S.A.J.); daviddelatorreduque@gmail.com (D.D.T.); liliansa@usp.br (L.R.M.S.); ajpferr@usp.br (A.J.P.F.);

[2] Inter-units Program in Biotechnology, University of São Paulo, Av. Prof. Lineu Prestes, 2415, 05508-000, São Paulo, Brazil;

[3] Pole Alimentos Ltda, Uberlândia, MG, Brazil; patriciacalixtovet@yahoo.com.br (P.C.P.);

[4] Department of Neurobiology, Duke University, Duke University Road, Durham, NC, USA; ac699@duke.edu (A.B.C-S.);

[5] LABIGEN, Laboratory of Molecular Biology and Genetics, Quito EC170521, Ecuador

[6] Department of Anatomy, Biomedical Sciences Institute, University of São Paulo, Av. Prof. Lineu Prestes, 2415, 05508-270, São Paulo, SP, Brazil.; so36ma@usp.br (S.R.Y.A.);

[7] Department of Surgery, School of Veterinary Medicine, University of São Paulo, Av. Prof. Dr. Orlando M. Paiva, 87, 05508-270, São Paulo, SP, Brazil; roseeli@usp.br (R.E.G.R.);

* Correspondence: ajpferr@usp.br; Tel.: +55-11-3091-1352

viruses

MDPI

Table S1. Zootechnical performance data of Flock USP-1408. Weekly Egg Breeding Performance and Mortality.

				EGG BREEDING PERFORMANCE							MORTALITY	
Week	Quantity of Hens	Quantity of Roosters	Sex Ratio	Weekly Egg Quantity	Cumulative Egg Quantity	Observed Hen-Day Egg Production (%)	Weekly Hatched Chicks	Cumulative Hatched Chicks	Observed Hatching Eggs (%)	Observed Cumulative Eggs-Hen Production	Observed Cumulative Hen Mortality (%)	Observed Cumulative Rooster Mortality (%)
**W**	**Hen**	**Rooster**	**SR**	**WEQ**	**CEQ**	**OHDEP**	**WHC**	**CHC**	**OHE**	**OCEHP**	**OCHM**	**OCRM**
25	15993	1680	10,5	15035	15933	13,4	11171	11171	65,0	0,99	0,2	0,4
26	15911	1668	10,5	50910	66843	45,7	38509	49680	73,3	4,15	0,7	1,1
27	15835	1650	10,4	82244	149087	74,2	67747	117427	81,0	9,27	1,2	2,2
28	15786	1641	10,4	93423	242510	84,5	68609	186036	86,7	15,07	1,5	2,7
29	15762	1625	10,3	97201	339711	88,1	78466	264502	88,0	21,11	1,6	3,7
30	15707	1620	10,3	97883	437594	89,0	95132	359634	90,0	27,20	1,8	4,0
31	15653	1584	10,1	98308	535902	89,7	86221	445855	91,3	33,31	2,2	6,3
32	15635	1576	10,1	98085	633987	89,6	85052	530907	91,0	39,40	2,3	6,8
33	15607	1565	10,0	96948	730935	88,7	82309	613216	90,0	45,43	2,5	7,5
34	15584	1555	10,0	93418	824353	85,6	67833	681049	89,7	51,23	2,6	8,1
35	15571	1583*	10,2	94447	918800	86,7	82935	763984	91,5	57,10	2,7	8,3
36	15562	1577	10,1	94254	1013054	86,5	83161	847145	90,5	62,96	2,8	8,7
37	15544	1575	10,1	94543	1107597	86,9	83849	930994	91,1	68,84	2,9	8,8
38	15535	1661*	10,7	94115	1201712	86,6	81875	1012869	89,1	74,69	3,0	10,2
39	15524	1636	10,5	93612	1295324	86,1	80286	1093155	88,6	80,50	3,0	10,5
40	15483	1626	10,5	91987	1387311	84,9	77835	1170990	88,1	86,22	3,3	11,1
41	15471	1622	10,5	90315	1477626	83,4	80478	1251468	87,4	91,84	3,4	11,4
42	15465	1618	10,5	89793	1567419	83,0	74891	1326359	85,6	97,42	3,4	11,6
43	15454	1601	10,4	89527	1656946	82,8	73932	1400291	86,1	102,98	3,5	12,7

44	15447	1593	10,3	88266	1745212	81,6	74061	1474352	86,2	108,47	3,5	13,2
45	15428	1585	10,3	86210	1831422	79,8	71234	1545586	84,6	113,82	3,6	13,7
46	15417	1571	10,2	84962	1916384	78,7	68658	1614244	84,0	119,10	3,7	14,5
47	15406	1570	10,2	83958	2000342	77,9	77508	1691752	82,7	124,32	3,8	14,6
48	15384	1562	10,2	84252	2084594	78,2	52304	1744056	80,8	129,56	3,9	15,1
49	15377	1557	10,1	82394	2166988	76,6	63364	1807420	79,1	134,68	4,0	15,4
50	15370	1484	9,7	81309	2248297	75,6	59927	1867347	75,8	139,73	4,0	15,6
51	15358	1478	9,6	79993	2328290	74,4	58262	1925609	73,5	144,70	4,1	16,0
52	15339	1473	9,6	79548	2407838	74,1	53382	1978991	70,8	149,65	4,2	16,4
53	15318	1847*	12,1	76426	2484264	71,3	50255	2029246	68,6	154,40	4,4	17,2
54	15286	1391	9,1	74030	2558294	69,2	47718	2076964	66,6	159,00	4,6	18,0
55	15252	1378	9,0	77457	2635751	72,6	48451	2125415	64,6	163,81	4,8	18,7
56	15223	1361	8,9	75015	2710766	70,4	46359	2171774	62,8	168,48	5,0	19,7
57	15191	1342	8,8	74394	2785160	70,0	43578	2215352	62,0	173,10	5,2	20,7
58	15158	1326	8,7	72167	2857327	68,0	41228	2256580	60,1	177,58	5,4	21,6
59	15120	1297	8,6	71432	2928759	67,5	34677	2291257	57,8	182,02	5,7	23,0
60	15090	1297	8,6	70477	2999236	66,7	39005	2330262	57,6	186,40	5,9	23,3
61	15034	1291	8,6	70245	3069481	66,8	31547	2361809	56,1	190,77	6,2	23,6
62	14997	1286	8,6	68055	3137536	64,8	35313	2397122	54,5	195,00	6,5	23,9
63	14954	1277	8,5	68314	3205850	65,3	34070	2431192	52,4	199,24	6,8	24,4
64	14495	1275	8,8	28912	3234762	65,3	16566	2447758	52,7	201,04	10,0	25,1

* Week that included a routine restocking of animals to regularize the SR.

viruses

MDPI

Table S2. Zootechnical performance data of Flock USP-1484. Weekly Egg Breeding Performance and Mortality.

				EGG BREEDING PERFORMANCE							MORTALITY	
Week	Quantity of Hens	Quantity of Roosters	Sex Ratio	Weekly Egg Quantity	Cumulative Egg Quantity	Observed Hen-Day Egg Production (%)	Weekly Hatched Chicks	Cumulative Hatched Chicks	Observed Hatching Eggs (%)	Observed Cumulative Eggs-Hen Production	Observed Cumulative Hen Mortality (%)	Observed Cumulative Rooster Mortality (%)
**W**	**Hen**	**Rooster**	**SR**	**WEQ**	**CEQ**	**OHDEP**	**WHC**	**CHC**	**OHE**	**OCEHP**	**OCHM**	**OCRM**
25	31813	3200	10,21	35965	38374	16,15	25678	25678	63,22	1,2	0,1	0,4
26	31665	3171	10,17	113084	151458	51,02	82990	108668	73,23	4,7	0,6	1,3
27	31547	3154	10,14	164959	316417	74,7	129351	238019	81,28	9,9	1,0	1,9
28	31489	3010	9,84	185085	501502	83,97	159754	397773	86,43	15,7	1,2	2,3
29	31454	2996	9,79	193238	694740	87,76	163924	561697	88,00	21,7	1,3	2,8
30	31432	2986	9,77	194270	889010	88,29	158250	719947	89,97	27,8	1,3	3,1
31	31393	3230*	10,23	195908	1084918	89,15	170049	889996	90,85	33,9	1,5	6,4
32	31348	3219	10,21	192465	1277383	87,71	168394	1058390	91,25	39,9	1,6	6,8
33	31260	3203	10,17	188014	1465397	85,92	165837	1224227	91,58	45,8	1,9	7,3
34	31227	3178	10,11	189432	1654829	86,66	182098	1406325	91,73	51,7	2,0	8,0
35	31195	3131	10,08	195201	1850030	89,39	171568	1577893	91,38	57,8	2,1	9,5
36	31165	3087	9,98	193462	2043492	88,68	167728	1745621	90,45	63,9	2,2	11,0
37	31150	3058	9,92	191083	2234575	87,63	168108	1913729	90,73	69,8	2,2	11,9
38	31136	2912	9,80	189809	2424384	87,09	166208	2079937	89,85	75,8	2,3	13,2
39	31115	2900	9,73	190833	2615217	87,62	166613	2246550	89,14	81,7	2,3	13,6
40	31092	2884	9,68	187732	2802949	86,26	159408	2405958	86,88	87,6	2,4	14,2
41	31072	2877	9,67	183410	2986359	84,32	153333	2559291	87,25	93,3	2,5	14,4
42	31047	2861	9,63	180328	3166687	82,97	146607	2705898	83,55	99,0	2,6	15,0
43	31032	2853	9,58	178010	3344697	81,95	151376	2857274	85,79	104,5	2,6	15,3

44	31004	2831	9,52	177184	3521881	81,64	147998	3005272	86,21	110,1	2,7	16,0
45	30973	2820	9,49	174479	3696360	80,48	143053	3148325	84,27	115,5	2,8	16,4
46	30930	2808	9,45	172386	3868746	79,62	140706	3289031	84,15	120,9	2,9	16,9
47	30908	2802	9,44	169500	4038246	78,34	138681	3427712	82,29	126,2	3,0	17,1
48	30881	2795	9,42	169046	4207292	78,20	133475	3561187	80,62	131,5	3,1	17,3
49	30851	2790	9,40	166987	4374279	77,32	129316	3690503	79,71	136,7	3,2	17,5
50	30821	2782	9,24	165728	4540007	76,82	122519	3813022	76,49	141,9	3,3	17,8
51	30791	2594	8,82	163246	4703253	75,74	115649	3928671	73,11	147,0	3,4	25,0
52	30752	2589	8,81	161530	4864783	75,04	111357	4040028	71,33	152,0	3,5	25,2
53	30712	2835*	10,17	160141	5024924	74,49	107870	4147898	70,06	157,0	3,7	29,7
54	30684	2829	9,18	156318	5181242	72,78	100201	4248099	66,74	161,9	3,7	29,9
55	30620	2822	9,16	154576	5335818	72,12	97593	4345692	65,23	166,7	4,0	30,1
56	30422	2809	9,14	152764	5488582	71,74	93087	4438779	63,88	171,5	4,6	30,6
57	30251	2783	9,08	149816	5638398	70,75	87330	4526109	61,54	176,2	5,2	31,5
58	30122	2759	9,02	145040	5783438	68,79	76372	4602481	58,29	180,7	5,6	32,4
59	30018	2737	8,94	143264	5926702	68,18	72957	4675438	58,93	185,2	6,0	33,2
60	29923	2718	8,92	141477	6068179	67,54	77231	4752669	57,17	189,6	6,3	33,9
61	29822	2697	8,89	139232	6207411	66,70	80028	4832697	55,60	194,0	6,6	34,7
62	29735	2680	8,87	136450	6343861	65,56	71495	4904192	55,06	198,3	6,9	35,3
63	29663	2668	8,84	132995	6476856	64,05	67357	4971549	52,92	202,4	7,1	35,8
64	29558	2653	8,92	132516	6609372	64,05	61789	5033338	50,73	206,5	7,5	36,3

* Week that included a routine restocking of animals to regularize the SR.

Table S3. Weighted Weekly Egg Breeding Performance and Mortality of Flocks (USP-1408 and USP-1484). Side-by-side comparison of Estimated versus Observed Data.

		EGG BREEDING PERFORMANCE						MORTALITY			
Week	Sex Ratio	Estimated* Hen-Day Egg Production (%)	Observed Hen-Day Egg Production (%)	Estimated* Cumulative Eggs-Hen Production	Observed Cumulative Eggs-Hen Production	Estimated* Hatching Eggs (%)	Observed Hatching Eggs (%)	Estimated* Cumulative Hen Mortality (%)	Observed Cumulative Hen Mortality (%)	Estimated* Cumulative Rooster Mortality (%)	Observed Cumulative Rooster Mortality (%)
**W**	**SR**	**EHDEP**	**OHDEP**	**ECEHP**	**OCEHP**	**EHE**	**OHE**	**ECHM**	**OCHM**	**ECRM**	**OCRM**
25	10,1	4,0	15,24	0,3	1,13	70,0	63,76	0,2	0,14	0,5	0,39
26	10,0	25,0	49,24	2,0	4,54	73,3	73,24	0,4	0,63	1,0	1,24
27	10,0	54,9	74,53	5,9	9,68	77,3	81,18	0,6	1,03	1,5	1,97
28	9,6	74,9	84,16	11,1	15,47	80,8	86,51	0,8	1,26	2,0	2,47
29	9,5	82,8	87,88	16,8	21,51	83,3	87,99	1,0	1,38	2,5	3,12
30	9,5	84,8	88,54	22,7	27,59	85,4	89,99	1,2	1,50	4,0	3,44
31	10,3	84,3	89,34	28,5	33,70	86,3	90,99	1,4	1,69	4,3	6,37
32	10,3	83,8	88,35	34,3	39,75	87,8	91,16	1,6	1,83	4,6	6,76
33	10,2	83,3	86,86	40,0	45,67	88,2	91,06	1,8	2,07	4,9	7,33
34	10,2	82,3	86,32	45,7	51,55	88,7	91,18	2,0	2,20	5,2	8,07
35	10,0	81,3	88,48	51,3	57,57	88,9	91,43	2,2	2,29	6,2	9,13
36	9,9	80,3	87,96	56,8	63,56	88,9	90,48	2,4	2,38	6,5	10,20
37	9,8	79,3	87,39	62,2	69,50	88,9	90,86	2,6	2,45	6,8	10,87
38	9,4	78,3	86,91	67,5	75,40	88,9	89,60	2,8	2,50	7,1	12,10
39	9,3	77,3	87,13	73,8	81,32	88,9	88,96	3,0	2,57	7,4	12,50
40	9,3	76,3	85,80	78,0	87,13	88,7	87,29	3,2	2,70	8,9	13,08
41	9,3	75,0	84,02	83,1	92,83	88,5	87,32	3,4	2,77	9,2	13,32
42	9,2	73,7	82,97	88,0	98,45	88,3	84,24	3,6	2,83	9,5	13,76
43	9,2	72,4	82,22	92,9	104,01	88,1	85,90	3,8	2,89	9,8	14,32
44	9,1	71,1	81,64	97,7	109,53	87,9	86,20	4,0	2,97	10,1	15,00
45	9,1	69,9	80,26	102,4	114,95	87,6	84,39	4,2	3,07	11,6	15,43
46	9,1	68,7	79,32	107,0	120,30	87,3	84,10	4,4	3,19	12,1	16,02
47	9,1	67,5	78,18	111,5	125,57	87,0	82,45	4,6	3,26	12,6	16,18
48	9,1	66,3	78,21	116,0	130,84	86,6	80,68	4,8	3,37	13,1	16,52
49	9,0	65,1	77,07	120,3	136,03	86,0	79,52	5,0	3,45	13,6	16,75

50	9,0	63,9	76,40	124,6	141,16	85,4	76,25	5,2	3,53	15,1	17,04
51	8,4	62,7	75,30	128,7	146,22	84,8	73,23	5,4	3,63	15,6	21,77
52	8,4	61,5	74,72	132,8	151,23	84,1	71,15	5,6	3,75	16,1	22,01
53	9,2	60,3	73,42	136,8	156,15	83,4	69,58	5,8	3,89	16,6	24,76
54	9,2	59,1	71,58	140,7	160,94	82,7	66,70	6,0	4,02	17,1	25,97
55	9,2	57,9	72,26	144,5	165,77	81,9	65,02	6,2	4,23	18,6	26,39
56	9,2	56,7	71,29	148,2	170,51	81,0	63,52	6,4	4,73	19,1	27,03
57	9,2	55,5	70,49	151,8	175,16	80,1	61,69	6,6	5,18	19,6	28,01
58	9,2	54,3	68,53	155,4	179,68	79,2	58,94	6,8	5,54	20,1	28,89
59	9,1	53,1	67,95	158,9	184,14	78,3	58,57	7,0	5,85	20,6	29,93
60	9,1	51,9	67,27	162,2	188,55	77,3	57,31	7,2	6,13	22,1	30,46
61	9,0	50,7	66,71	165,5	192,90	76,3	55,75	7,4	6,48	22,6	31,09
62	9,0	49,5	65,31	168,7	197,16	75,2	54,88	7,6	6,76	23,1	31,60
63	9,0	48,3	64,46	171,9	201,34	74,1	52,75	7,8	7,02	23,6	32,08
64	9,0	47,1	52,35	174,9	204,73	72,8	51,14	8,0	8,31	24,1	32,67

* Estimated data according with the performance standards guidelines for the lineage (Ross 308 AP – AP95).

Available at: https://pt.aviagen.com/assets/Tech_Center/BB_Foreign_Language_Docs/Portuguese/Ross308AP-PS-PO-PT-2017.pdf

SR = Sex Ratio (male/female ratio). OHDEP = Observed Hen-Day Egg Production (daily egg quantity/total quantity of hens).

OCEHP = Observed Cumulative Eggs-Hen Production (weighted). OHE = Observed Hatching Eggs (weighted).

OCHM = Observed Cumulative Hen Mortality (weighted). OCRM = Observed Cumulative Rooster Mortality (weighted).

Table S4. Avipoxvirus strains with available complete genome selected for sequence comparison.

Strain	Species	Date	Source/host	Country	Genome Length (base pairs)	GenBank Accession #	Reference
FPVUS	Fowlpox virus	1999	*Gallus gallus* embryo	USA	288539	AF198100	[28]
FP9, HP-438/Munich	Fowlpox virus (att)	1966	*Gallus gallus* embryo	Poland	266145	AJ581527	[29]
PSan92	Penguinpox virus	1992	*Spheniscus demersus*	South Africa	306862	KJ859677	[30]
FeP2	Pigeonpox virus	2011	*Columba livia*	South Africa	282356	KJ801920	[30]
TKPV-HU1124/2011	Turkeypox virus	2011	*Meleagris gallopavo*	Hungary	188534	KP728110	[17]
SWPV-1	Shearwaterpox virus	2016	*Ardenna carneipes*	Australia	326929	KX857216	[4]
FGPVKD09	Flamingopox virus	2008	*Phoenicoparrus minor*	South Africa	293123	MF678796	[31]
16117_scab_170512	Fowlpox virus	2016	*Gallus gallus*	France	288539	MF766432	[32]
16069_trachea_170323	Fowlpox virus	2016	*Gallus gallus*	France	288539	MF766431	[32]
16055_trachea_170512	Fowlpox virus	2016	*Gallus gallus*	France	288539	MF766430	[32]
FWPV-SD15-670.2	Fowlpox virus	2015	*Meleagris gallopavo merriami*	USA	295938	MH734528	[10]
FWPV-SD15-670.1	Fowlpox virus	2015	*Meleagris gallopavo merriami*	USA	288196	MH719203	[10]
FWPV-MN00.1	Fowlpox virus	2000	*Meleagris gallopavo merriami*	USA	291057	MH709125	[10]
FWPV-MN00.2	Fowlpox virus	2000	*Meleagris gallopavo merriami*	USA	298798	MH709124	[10]
282E4	Fowlpox vírus	2011	*Gallus gallus*	China	308827	MG702259	Unpublished
MPPV 18-0641	Magpiepox virus	2018	*Gymnorhina tibicen*	Australia	293226	MK903864	[5]
NX10	Fowlpox virus	2010	*Gallus gallus*	China	282402	KX196452	Unpublished

Table S5. Discrepancies analysis of primers for specific detection of clade E avipoxvirus.

Target Gene	Primer	Size (bp)	Sites in alignment*	Range Identities	Range Differences	Max Identity
ORF49	qEPox49-F	22	27	2 – 15	10 – 16	55.56%
ORF49	qEPox49-R	20	28	6 – 10	18 – 22	35.71%
ORF114	qEPox114-F	20	20	5 – 9	7 – 12	53.33%
ORF114	qEPox114-R	20	20	5 – 7	13 – 15	35.00%
ORF159	qEPox159-F	21	21	8 – 16	5 – 13	76.19%
ORF159	qEPox159-R	21	33	7 – 15	6 – 26	71.43%

* Including Gaps.

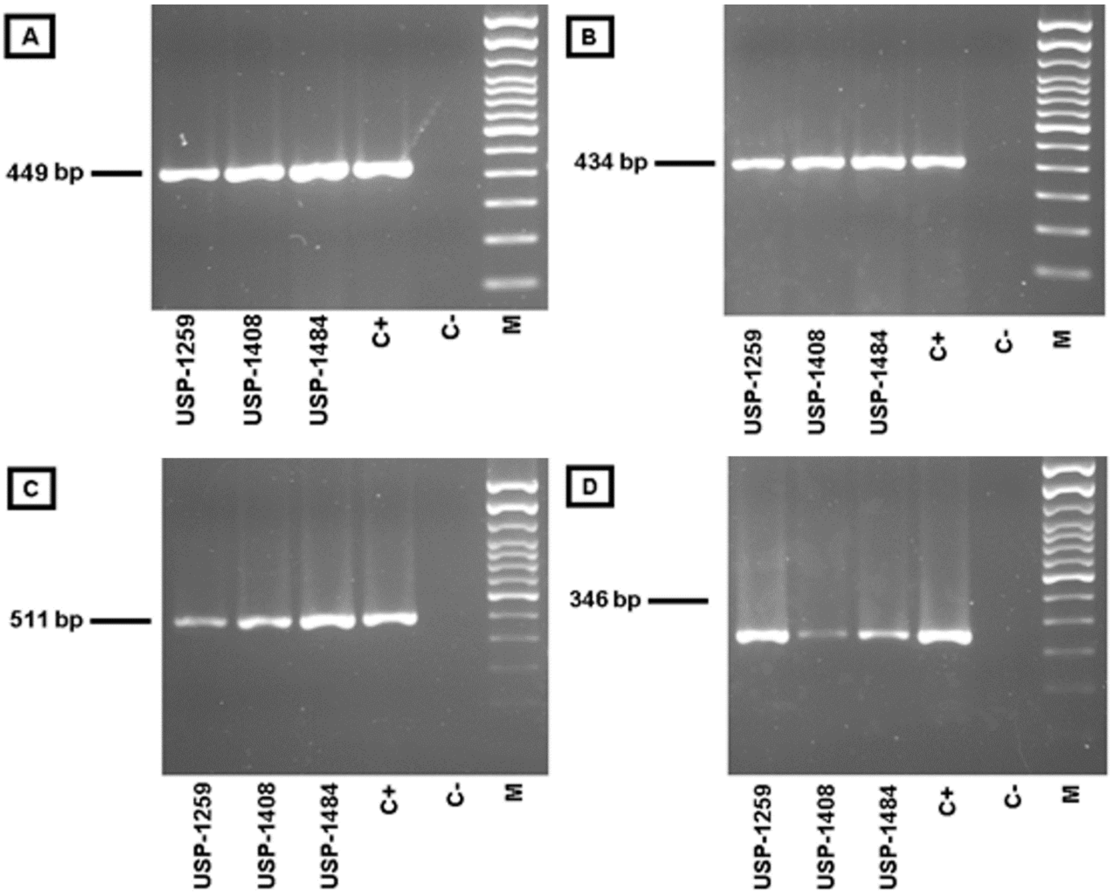

Figure S1. Electrophoresis for external primers products for **A.** ORF49, **B.** ORF114, **C.** ORF159, **D.** Beta-actin gene. C+ = positive control, C- = negative control, M = molecular size marker (100 bp).

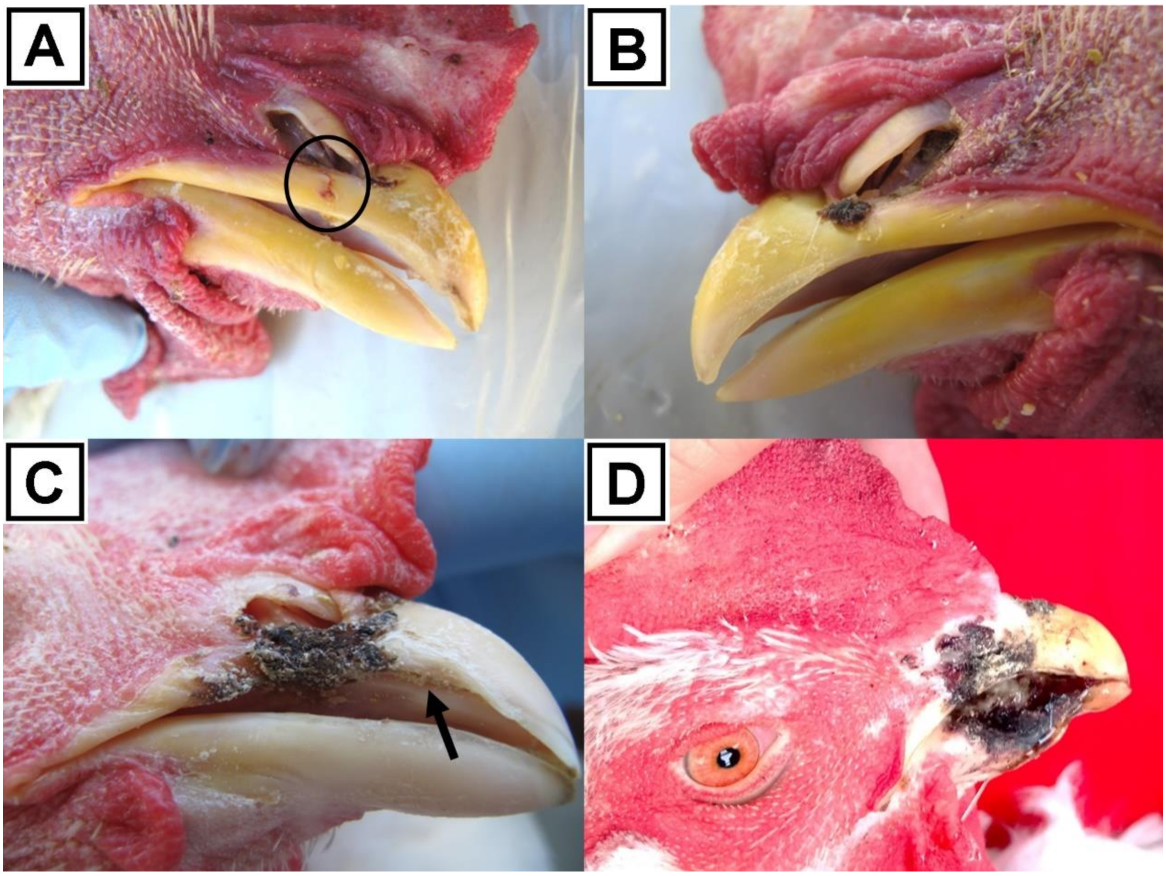

Figure S2. Clinical signs observed in roosters and hens of broiler breeders by different degrees of severity. **A.** Lateral side of the upper corneous beak showing a crack with a hemorrhagic crust (circle). **B.** Caudolateral face of the upper corneous beak presents a blackened necrotic crust in the entrance as well as in the caudal corner of the nostril. **C.** Partial loss of the caudolateral portion of the upper corneous beak showing a blackish and irregular crust that extends ventrally and rostrally to the right nostril. Note the irregularity of the sharp ventral border (arrow). **D.** Extensive necrosis of the caudolateral face of the upper and lower beak, whose blackish hemorrhagic crust covers the entrance to the right nostril.

viruses MDPI

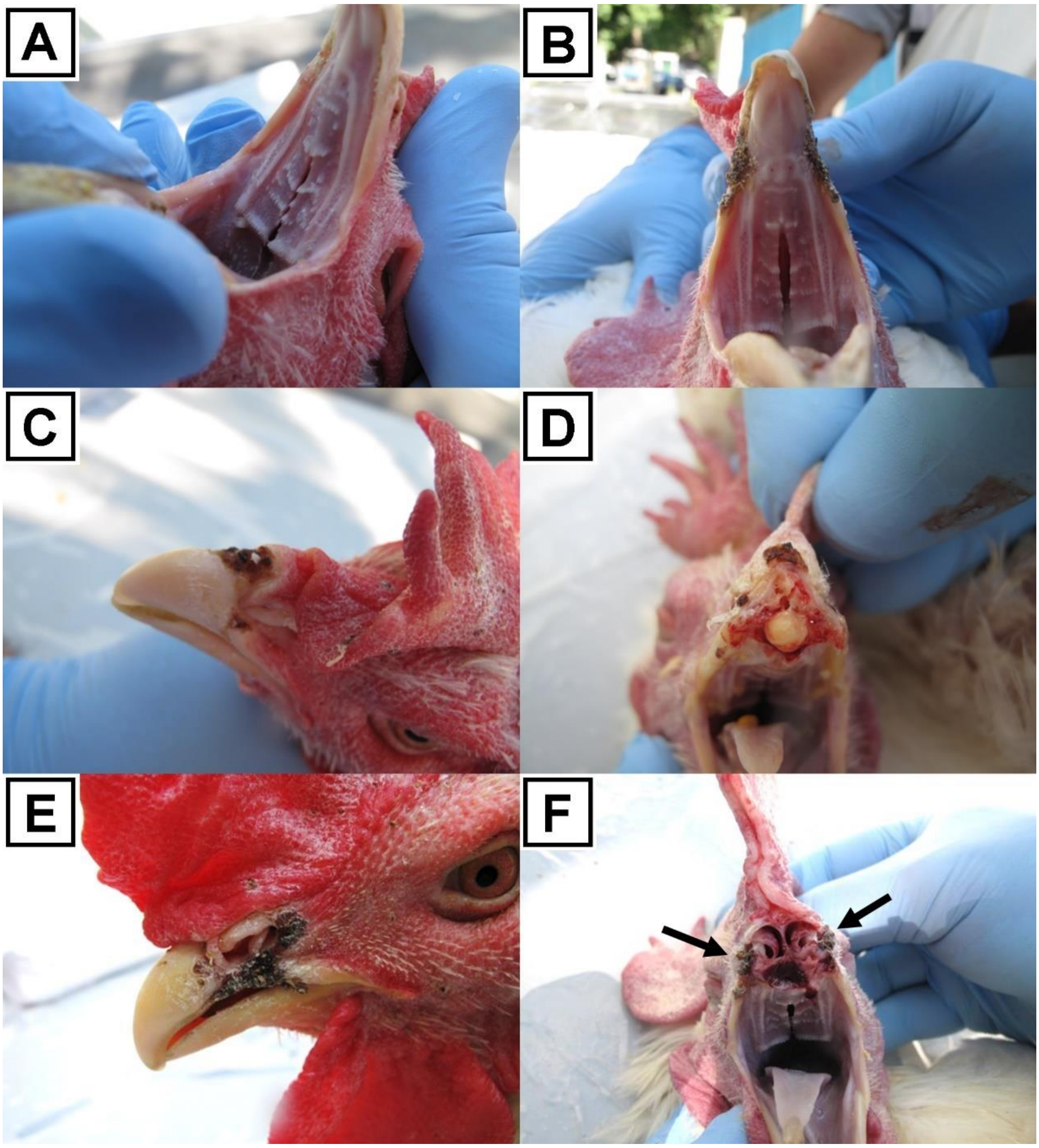

Figure S3. Beak lesions of broiler breeders varying from erosions in nostrils to profound damage of choanas. **A.** Erosion and irregularity of the ventromedial edge of the upper beak. **B.** Erosion and irregularity covered by a blackish hemorrhagic crust on the ventromedial border and part of the hard palate. **C.** Hemorrhagic crust compromising between the culmen of the beak and the rostral part of the nostril. Note the irregularity of the left caudolateral surface of the upper beak. **D.** Caudodorsal region of the culmen in the rostral view showing irregularity of the culmen and hemorrhagic crust. **E.** Left caudolateral face of the upper corneous beak showing a blackish, necrotic crusted lesion that compromises the ventromedial border and caudal portion of the nostril. **F.** Caudal region of the upper corneous beak in section, where bilateral symmetrical lesions are observed on the lateral edges, which are irregular covered by hemorrhagic crust and the rostral nasal chamber with hemorrhagic crust (arrows).

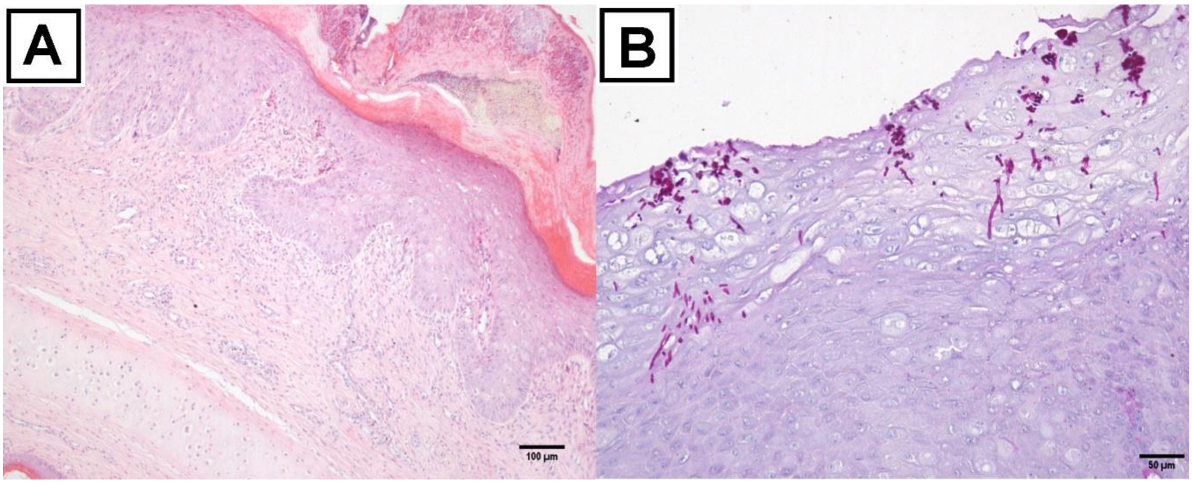

Figure S4. Photomicroscopy of irregular epithelium of the upper beak.**A.** Caudolateral region of the upper beak close to the nasal cartilage. Note the irregular epithelium, exuberant crust covering the epithelium and cartilage in the ventral portion. **B.** Zoom in the superficial epithelium of the upper beak, showing hyphae infiltrating between the keratinocytes. Periodic acid-Schiff (PAS) staining.

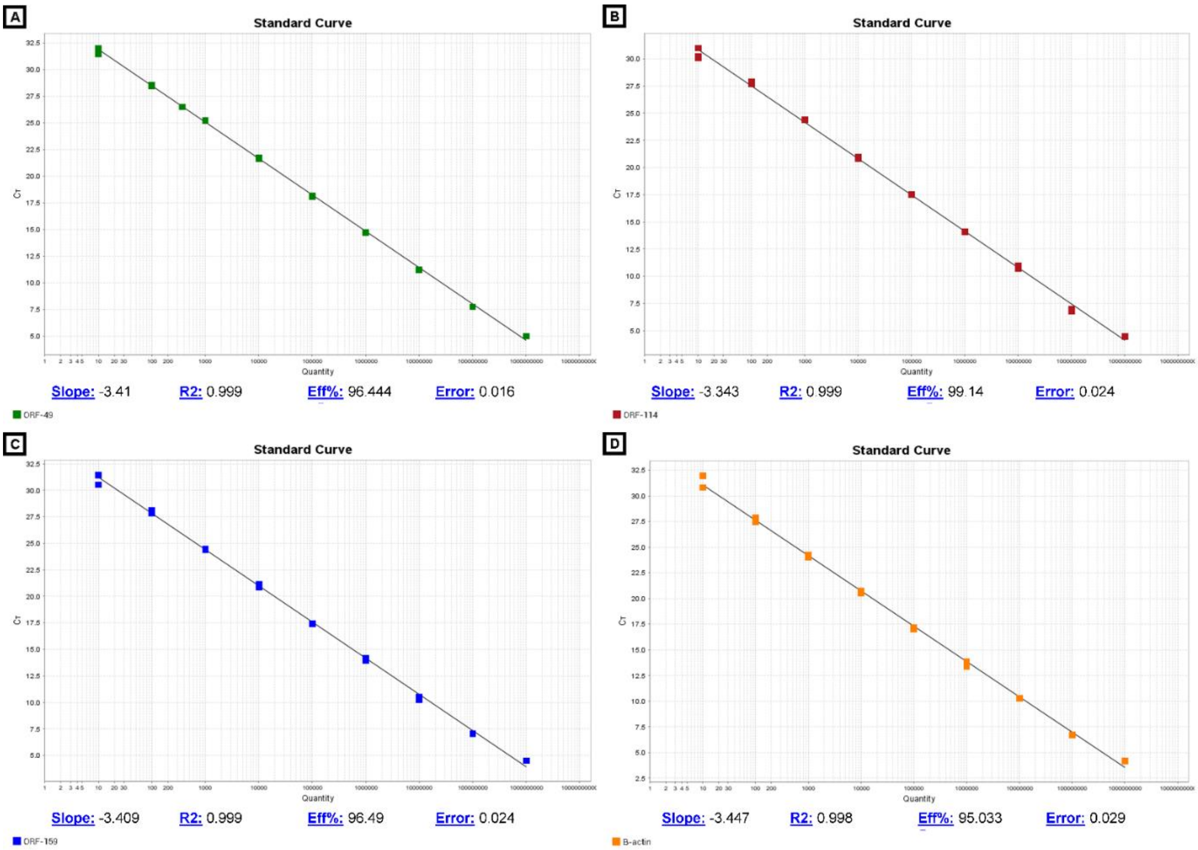

Figure S5. Standard curve plots for qPCR assays developed for detection and quantification of Clade E Avipoxvirus. **A.** ORF49, **B.** ORF114, **C.** ORF159, **D.** Beta-actin gene.

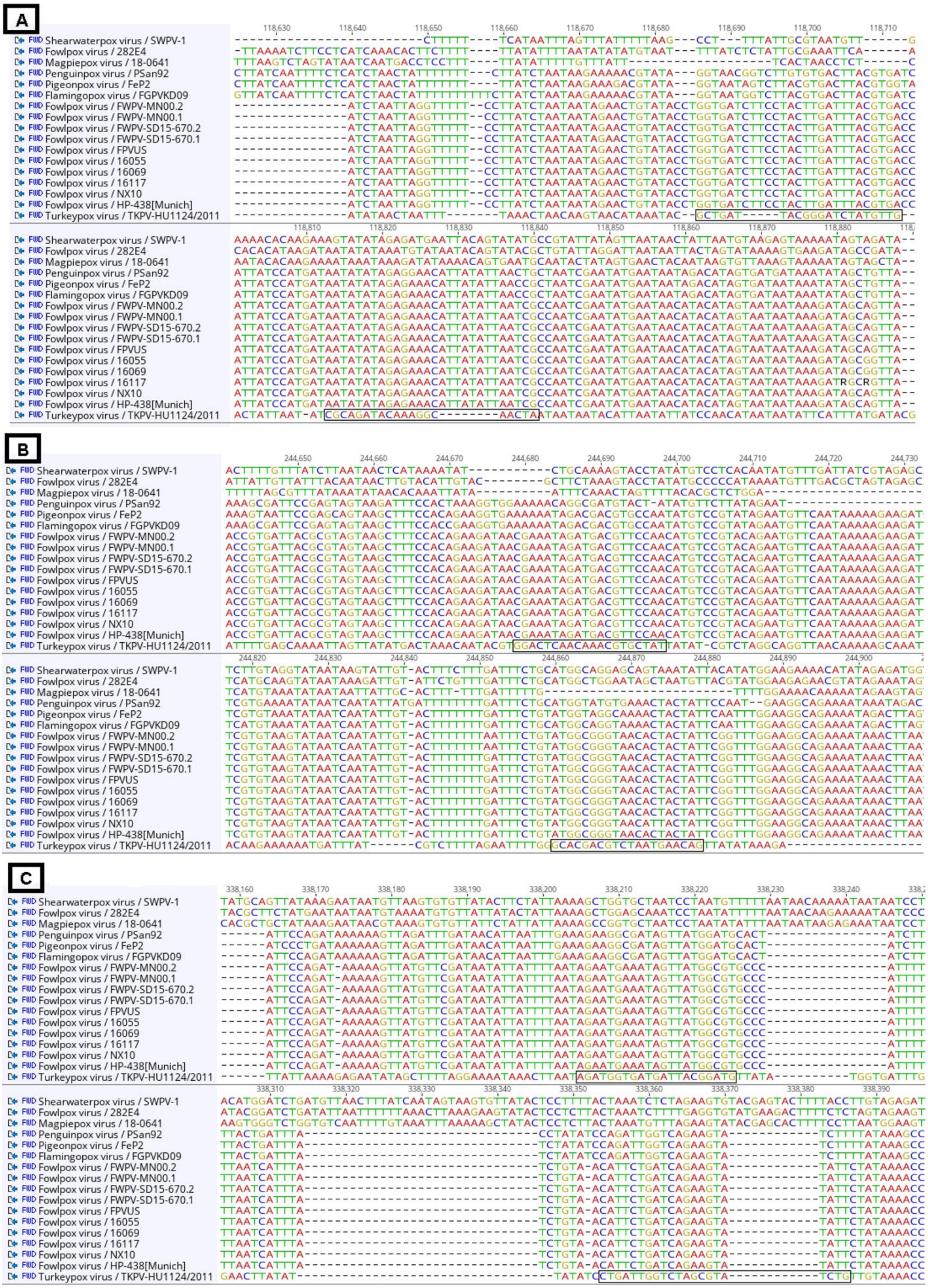

Figure S6. Target regions of primers for **A.** ORF49, **B.** ORF114, **C.** ORF159, in avipoxvirus genome alignment designed for specific amplification of clade E avipoxvirus. Nucleotide sequences used for primer design were enclosed in the rectangles.
